# Biologically relevant transfer learning improves transcription factor binding prediction

**DOI:** 10.1186/s13059-021-02499-5

**Published:** 2021-09-27

**Authors:** Gherman Novakovsky, Manu Saraswat, Oriol Fornes, Sara Mostafavi, Wyeth W. Wasserman

**Affiliations:** 1 Centre for Molecular Medicine and Therapeutics, BC Children’s Hospital Research Institute, Vancouver, BC V5Z 4H4 Canada; 2 Department of Medical Genetics, University of British Columbia, Vancouver, BC V6H 3 N1 Canada; 3 Department of Statistics, University of British Columbia, Vancouver, BC V6T 1Z4 Canada; 4 Canadian Institute for Advanced Research, CIFAR AI Chair, and Child and Brain Development, Toronto, ON M5G 1 M1 Canada

**Keywords:** Transfer learning, Deep learning, Transcription factor binding prediction, Model interpretation

## Abstract

**Background:**

Deep learning has proven to be a powerful technique for transcription factor (TF) binding prediction but requires large training datasets. Transfer learning can reduce the amount of data required for deep learning, while improving overall model performance, compared to training a separate model for each new task.

**Results:**

We assess a transfer learning strategy for TF binding prediction consisting of a pre-training step, wherein we train a multi-task model with multiple TFs, and a fine-tuning step, wherein we initialize single-task models for individual TFs with the weights learned by the multi-task model, after which the single-task models are trained at a lower learning rate. We corroborate that transfer learning improves model performance, especially if in the pre-training step the multi-task model is trained with biologically relevant TFs. We show the effectiveness of transfer learning for TFs with ~ 500 ChIP-seq peak regions. Using model interpretation techniques, we demonstrate that the features learned in the pre-training step are refined in the fine-tuning step to resemble the binding motif of the target TF (i.e., the recipient of transfer learning in the fine-tuning step). Moreover, pre-training with biologically relevant TFs allows single-task models in the fine-tuning step to learn useful features other than the motif of the target TF.

**Conclusions:**

Our results confirm that transfer learning is a powerful technique for TF binding prediction.

**Supplementary Information:**

The online version contains supplementary material available at 10.1186/s13059-021-02499-5.

## Background

A subset of human DNA-binding transcription factors (TFs) control gene expression at the transcriptional level by recognizing and binding to specific sequence motifs within *cis*-regulatory regions known as TF binding sites (TFBSs) [1]. The disruption of TF genes and TFBSs is associated with rare genetic disorders [2, 3] and cancer [4, 5]. Therefore, delineating the regions to which TFs bind in the genome could indicate potential regulatory regions on which to focus analyses and help to broaden our understanding of how genes are regulated in health and disease.

Chromatin immunoprecipitation followed by sequencing (ChIP-seq) is an experimental assay that enables the identification of TF-bound regions in vivo at a resolution of a few hundred base pairs (bp) [6]. These regions, known as ChIP-seq peaks, are expected to be enriched for TFBSs. The ReMap database has compiled and uniformly reprocessed thousands of public ChIP-seq datasets [7, 8]. It provides access to millions of ChIP-seq peaks related to the binding of approximately 800 human TFs in 602 different human cell and tissue types. Based on ReMap, the UniBind database stores reliable TFBS predictions from four different computational models, including position weight matrices (PWMs; reviewed in [9]), for the ChIP-seq peaks of 231 human TFs in 315 different human cell and tissue types [10].

Despite large-scale data generation efforts by public consortia such as ENCODE [11], delineating the binding regions of each human TF in the genome remains incomplete. For instance, about 40% of human TFs have not been profiled by ChIP-seq, and only a few, such as CTCF, have been profiled extensively in multiple biological contexts (e.g., across a range of cell and tissue types). To complement data generation efforts, deep learning methods have become pervasive, as high quality and large-scale datasets have drastically improved their performance (reviewed in [12]). A large training dataset is fundamental to the success of deep learning methods; however, the amount of ChIP-seq data for the majority of human TFs, if available, is small. For example, of the human TFs stored in ReMap, 381 (47.6%) have been profiled in only one cell or tissue type, and 134 (16.7%) have less than 1000 annotated ChIP-seq peaks.

Transfer learning—reusing the information learned from a model developed for one task as the starting point for a model on a second different, but related, task—has been shown to reduce the amount of data required for training while improving overall model performance for diverse applications (reviewed in [13]). In biology, transfer learning has been successful in several areas, including: reconstructing gene regulatory networks [14–16]; modeling gene expression from single-cell data [17–20]; or predicting genomic features, including accessible regions [21], chromatin interactions [22], and TFBSs [23, 24].

The current approach to transfer learning in the field of computer vision consists of two steps: pre-training a model on a large dataset (e.g., ImageNet [25]) and fine-tuning the model weights to suit a task of interest. The rationale is that in the pre-training step, the model learns low-level image features such as lines or curves [26], which are generalizable to the downstream task. For TF binding prediction, this translates into pre-training a multi-task model with as much genomic data as possible to learn common DNA features (e.g., TF binding motifs), so that in the fine-tuning step, the downstream task can exploit these common features learned in the pre-training step while focusing on learning novel features specific to that task. It is often unclear what characteristics are responsible for the superior performance of transfer learning. For example, the learning process could reveal common motifs in regulatory regions, simpler DNA sequence composition properties (e.g., %GC content), or other features, some of which might be novel to our understanding of TF binding specificity. Gaining insights into how the learning process influences predictive performance would allow for a broader adoption of transfer learning in genomics.

In this study, we perform an in-depth assessment of transfer learning for TF binding prediction. We corroborate the findings of Zheng and colleagues that transfer learning improves model performance, especially for TFs with small training datasets [24]. We show that transfer learning can perform well even when training with as few as 50 ChIP-seq peaks. We demonstrate that the benefit of transfer learning is greater when pre-training with biologically relevant TFs. Using model interpretation techniques, we observe that the features learned in the pre-training step are refined in the fine-tuning step to resemble the motif of the target TF (i.e., the recipient of transfer learning in the fine-tuning step), and pre-training with biologically relevant TFs allows the model to learn useful features other than the motif of the target TF in the fine-tuning step, such as the motifs of cofactors. Our results advocate for a broader adoption of transfer learning in bioinformatics-related deep learning studies.

## Results

### A sparse matrix of TF binding events across accessible genomic regions

Deep learning-based TF binding prediction can be treated as a binary classification task wherein the ones and zeros (or positives and negatives) represent whether or not a TF binds to a genomic region. It is common to define the regions a TF binds to as the set of ChIP-seq peaks for that TF, with unbound regions being the ChIP-seq peaks from other TFs or randomly selected genomic regions. Leaving aside the intrinsic challenges associated with ChIP-seq data generation and peak-calling [27], there are limitations to adopting such definitions. For instance, many ChIP-seq peaks lack the consensus motif of the profiled TF [28], while others are the consequence of indirect or tethered binding events [29, 30], or appear in datasets for unusually high numbers of other TFs [31–33], i.e., they may be artifacts. While bound regions can be directly defined from the ChIP-seq data, the selection of unbound regions for use in deep learning models is more difficult. For example, the %GC content, which is often an important contributor to model performance both in vitro [34] and in vivo [24], varies between the set of peak regions of different TFs [35]. Moreover, some classes of TFs are special: pioneer TFs can bind to nucleosome regions, which have distinctive sequence characteristics [36] and are not usually bound by non-pioneer TFs [37]. Thus, it is suboptimal to define the set of unbound regions for a given TF based on the set of bound regions of other TFs. The alternative approach of using randomly selected genomic regions as unbound regions can result in the inclusion of a set of non-relevant regions, such as centromeres or telomeres. Furthermore, a region not bound by a TF in a certain cell or tissue type could be bound by that same TF in a different biological context. Due to the dependence of deep learning on high quality data [38], properties arising from one or more of the aforementioned limitations could result in improperly fitted models and, ultimately, mislead or inflate model performance.

**Fig. 1:**
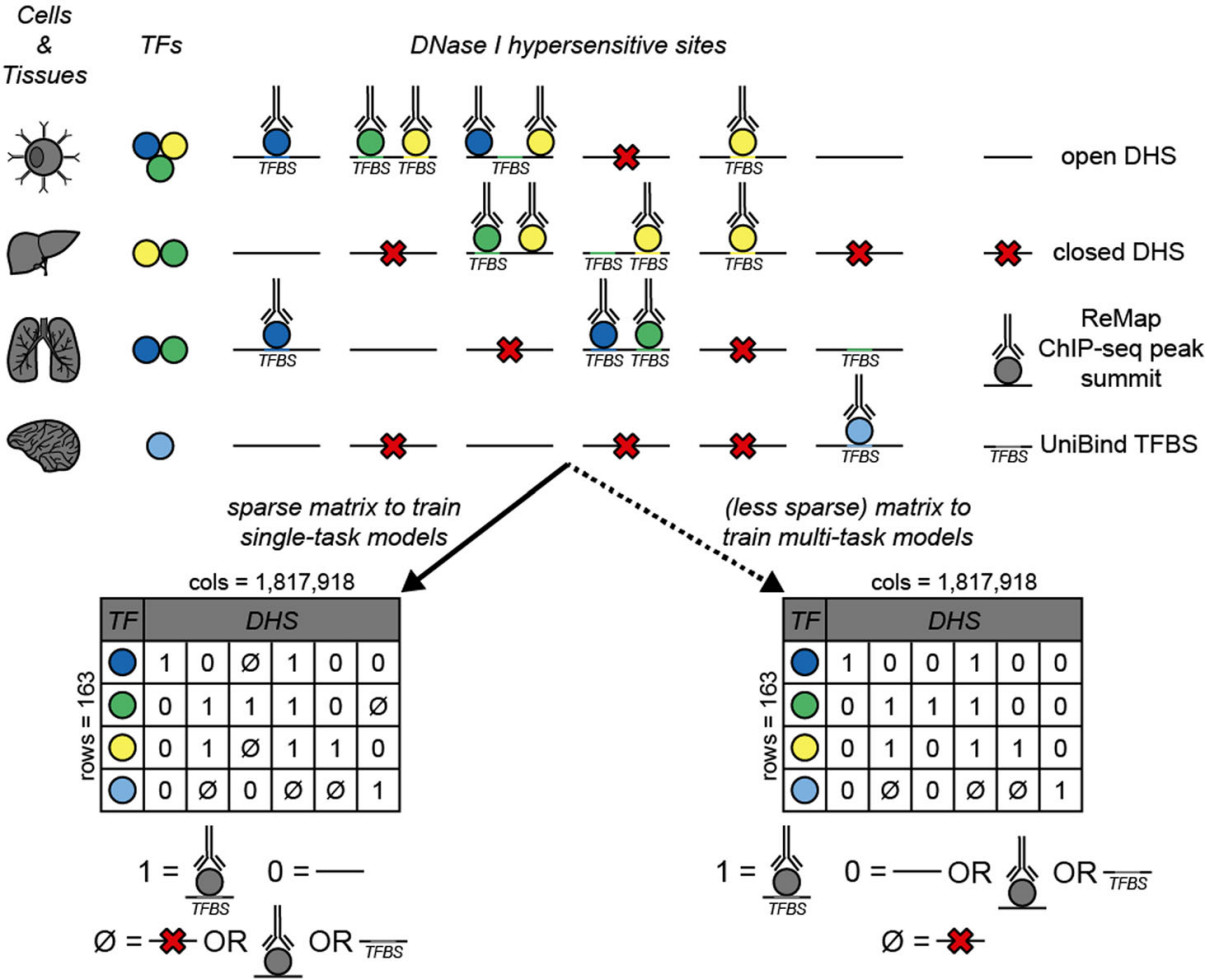
Two matrices of TF binding events across accessible genomic regions. The matrices, one more “sparse” for training single-task models and the other “less sparse” for training multi-task models, summarize the binding (from ReMap ChIP-seq peaks and UniBind TFBSs) of 163 TFs to 1,817,918 DHSs across 52 cell and tissue types, with rows representing TFs and columns representing DHSs. For each TF-DHS pair in the matrix, a “1” indicates that, according to both ReMap and UniBind, the TF binds to the DHS, and that the DHS is open (i.e., accessible), a “0,” that the DHS is open but that the TF does not bind to it, and a null sign (“∅”) indicates that there is insufficient evidence to assign a one or a zero, for instance, if the DHS is closed (i.e., not accessible). Both matrices have the same number of ones; however, they differ in the number of zeros and nulls. In the sparse matrix, TF-DHS pairs wherein the DHS is open and the TF binds to it according to either ReMap or UniBind data (not both) are assigned a null value; instead, in the less sparse matrix, they are assigned a zero value. DHS, DNase I hypersensitive site; TF, transcription factor; TFBS, TF binding site

To mitigate these limitations, which in turn could negatively impact on our assessment of transfer learning, we restricted the selection of bound and unbound regions to open regulatory regions of the genome. We constructed a sparse matrix describing the binding of 163 TFs to 1,817,918 200-bp of DNase I hypersensitive sites (DHSs) in a cell and tissue type-agnostic manner (Methods; Fig. 1). Each element in the matrix was defined by a specific TF-DHS pair and could take one of three values: “1” (i.e., bound) if the DHS was both accessible and bound by the TF in at least one cell or tissue type in common, “0” (i.e., not bound) if the DHS was accessible but not bound by the TF in any cell or tissue types in common, or “null” if the binding of the TF to the DHS could not be resolved (e.g., the TF had not been profiled in a cell or tissue type with available DHS data). The total number of ones, zeros, and nulls in the matrix was ~ 1.9 M, ~ 51.7 M, and ~ 242.6 M, respectively. In addition, the number of ones for each TF varied greatly, with 16 TFs having ≤ 500 bound regions (Table S1).

**Fig. 2:**
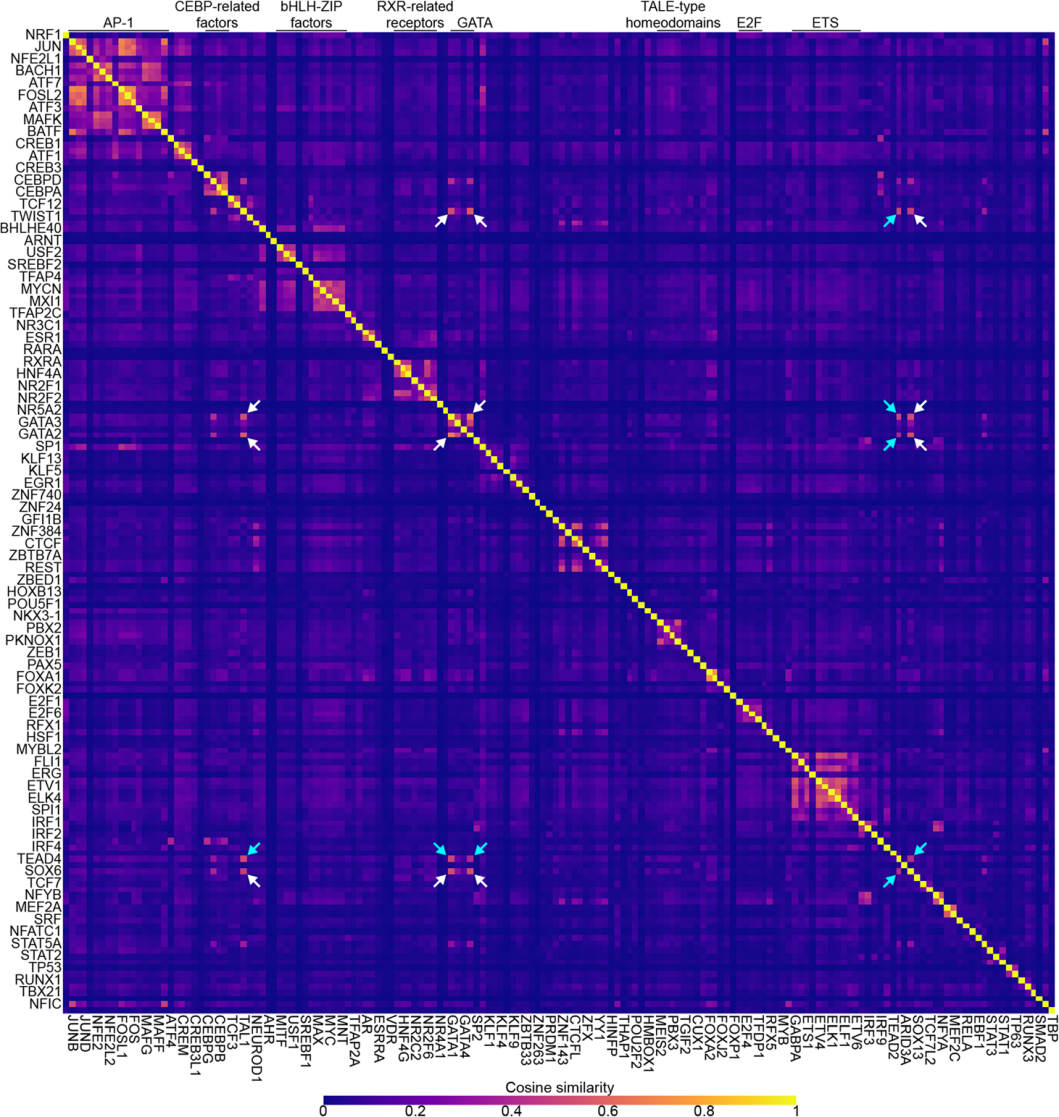
Heatmap of pairwise cosine similarities between the binding vectors of 162 TFs (SMAD3 is excluded because it had no bound regions). Homologous TFs from the same families form well-defined clusters along the heatmap’s diagonal. Highlighted with white arrows, an example of cooperativity for highly correlated TFs from different families, namely GATA1, GATA2, SOX6, and TAL1, which are required for erythropoiesis [40]. TEAD4 is also highly correlated with these four TFs (teal arrows), in accordance with previous literature [41]

The number of unresolved elements (> 80%) led to concerns regarding the sparsity of the matrix and whether it had captured known TF-TF functional associations present in ChIP-seq data [30]. We computed pairwise cosine similarities between the binding vectors of all TFs as a measure of correlation (Methods). To ease interpretation, TFs were sorted by their hierarchy in TFClass [39] and visualized on a heatmap (Fig. 2). As expected, TFs with shared DNA-binding mechanisms (hereafter referred to as binding modes), such as homologs or overlapping members of dimeric complexes, formed well-defined clusters along the heatmap diagonal. In contrast, away from the diagonal of the heatmap, correlated points could indicate the cooperative binding between TFs from different families, such as the erythropoietic TFs GATA1, GATA2, SOX6, and TAL1 [40], highlighted on the heatmap using white arrows. In agreement with previous literature [41], TEAD4 was also highly correlated with these four TFs (teal arrows). To further support the presence of cooperative binding in the TF binding matrix, we compared the binding vector similarities of TF-TF interacting pairs with different degrees of confidence from STRING [42]. On average, the binding vectors of the “highest confidence” TF pairs exhibited a higher degree of similarity than the rest (0.141 vs. 0.063; Welch *t* test, *p* value = 6.46e−09). We concluded that the TF binding matrix captured TF cooperativity and thus was well suited for our assessment of transfer learning.

### Transfer learning improves TF binding prediction

**Fig. 3:**
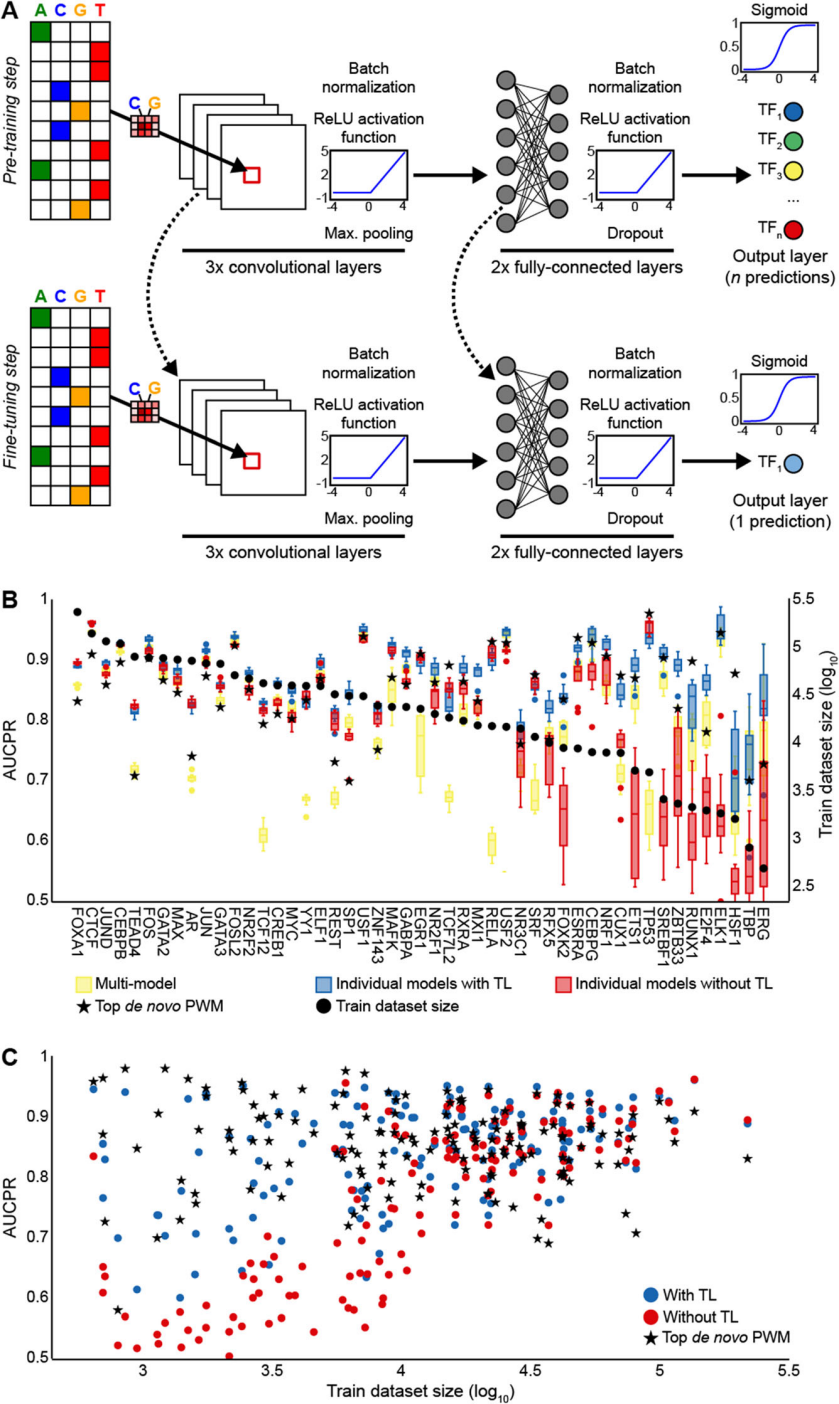
Transfer learning improves TF binding prediction. **A** The transfer learning strategy used in this study consists of two steps: pre-training a multi-task CNN with multiple TFs (top) and fine-tuning a single-task CNN, initialized with the weights of the pre-trained multi-task CNN, for one TF (bottom). The CNN architecture is similar to those of Basset [57] and AI-TAC [58]: three convolutional layers, each with ReLU activation, batch normalization, and max-pooling, followed by two fully connected layers and one output layer. Models were trained with one-hot encoded, 200-bp long DNA sequences, and their reverse complements. **B** Performance of individual models trained with (blue boxes) and without (red boxes) transfer learning for the 50 TFs from the pre-training step. The performance of each TF on the multi-model is provided as baseline (yellow boxes). **C** Quantification of the effect of training dataset size on model performance. TFs (dots) are plotted with respect to the size of their training dataset (*x*-axis) and performance of their individual models trained with (blue) and without (red) transfer learning (*y*-axis). The performance of de novo PWMs (black stars) is provided as a baseline for each TF. AUCPR, area under the precision-recall curve; CNN, convolutional neural network; PWM, position weight matrix; ReLU, rectified linear units; TF, transcription factor; TL, transfer learning

Zheng and colleagues recently applied transfer learning to identify distinctive contextual sequence characteristics of bound and unbound instances of TF motifs [24]. Their transfer learning framework, named AgentBind, included a pre-training step, wherein a multi-task model (hereafter referred to as multi-models) was trained on the DeepSEA dataset [43], and a fine-tuning step, wherein single-task models (hereafter referred to as individual models) for 38 individual TFs of the GM12878 cell line were trained after previous initialization of the convolutional layers with weights from the multi-model. AgentBind outperformed models trained from scratch (i.e., without transfer learning), particularly for TFs with little ChIP-seq data. To corroborate these results, we implemented a similar two-step transfer learning strategy for TF binding prediction (Methods; Fig. 3A); however, in the fine-tuning step, both the convolutional and fully connected layers (except the output layer) of individual models were initialized with the multi-model weights, after which the training continued at a lower learning rate. We trained a multi-model using the 50 TFs that maximized the number of common resolved regions (i.e., not null) in the TF binding matrix. Moreover, we trained individual models, with and without transfer learning, for 49 of these TFs; ARNT was excluded because it had < 250 resolved regions left (i.e., non-overlapping with multi-model regions). Transfer learning significantly improved model performance for 39 (79.6%) TFs (Fig. 3B; Wilcoxon signed-rank test, *p* value = 1.91e−07), especially for TFs with small training datasets, which is in concordance with the results from AgentBind. For the 10 cases where transfer learning was detrimental, the differences in model performance compared to training from scratch were small (ΔAUCPR < 0.025; Table S2).

The pre-training step included data for all TFs, so for a more direct comparison, we repeated the previous experiment with 99 additional TFs that had at least 250 bound regions in the TF binding matrix and, importantly, had not been used to train the multi-model. Again, transfer learning significantly improved model performance (Fig. 3C; Wilcoxon signed-rank test, *p* value = 2.92e−13) and was more beneficial for TFs with small training datasets. Among the most notable examples was ATF4, with only 557 bound regions, which achieved an AUCPR of 0.941 (compared to 0.571 when training from scratch). However, transfer learning was detrimental for 15 (17.1%) TFs (Table S2). By design of the TF binding matrix, the bound regions of each TF contained the motif of that TF, while the unbound regions could or could not contain the TF motif. This could make their classification too simple a task for the model. We therefore generated de novo PWMs for each TF to provide a more reliable baseline (Methods). Overall, transfer learning performance was comparable to that of de novo PWMs (0.845 vs. 0.856; Wilcoxon signed-rank test, *p* value = 0.667, NS); however, de novo PWMs outperformed transfer learning for TFs with small training datasets (Fig. 3C). Noteworthy, transfer learning reduced the variation in model performance resulting from different data splits (i.e., the random assignment of data to different training, validation and test sets), thereby increasing the overall robustness of the training process (Fig. S1).

**Fig. 4:**
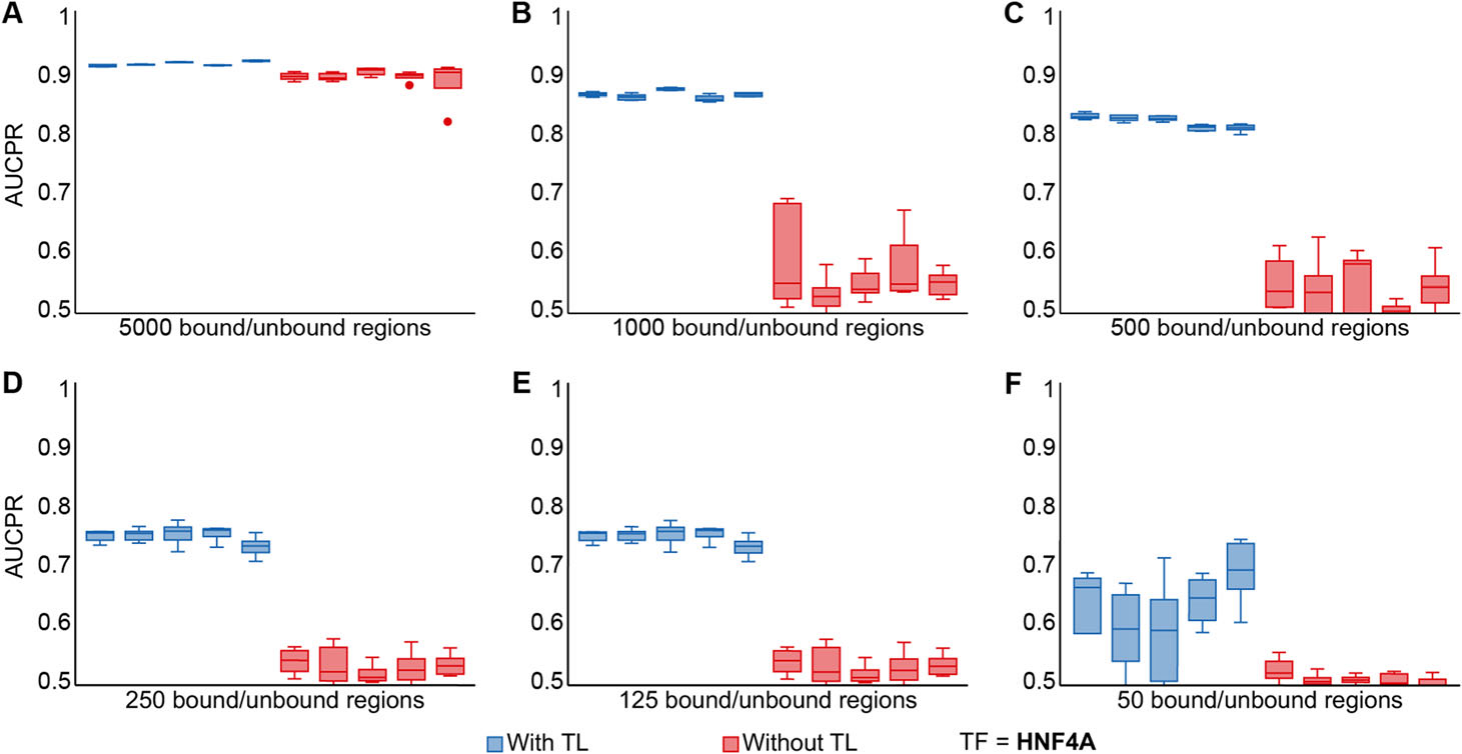
Transfer learning performs well for TFs with small training datasets. Performance of HNF4A models trained with (blue boxes) and without (red boxes) transfer learning on 5000 (**A**), 1000 (**B**), 500 (**C**), 250 (**D**), 125 (**E**), and 50 (**F**), bound and unbound regions. Each model was trained five times with different random initializations to ensure the robustness of the results. AUCPR, area under the precision-recall curve; TF, transcription factor; TL, transfer learning

Driven by the observation that transfer learning benefited TFs with small training datasets in particular, we sought to explore the minimum training dataset size required for effective transfer learning. We focused on five TFs from five different families: HNF4A (a nuclear receptor), JUND (a basic leucine zipper), MAX (a basic helix-loop-helix factor), SPI1 (a tryptophan cluster factor), and SP1 (a C2H2 zinc finger). Of these, JUND, MAX, and SP1 were among the 50 TFs used to train the multi-model. For each TF, we trained individual models, with and without transfer learning, by randomly downsampling to 5000, 1,000, 500, 250, 125, and 50, bound and unbound regions from the TF binding matrix. We repeated the downsampling process five times to ensure the robustness of the results. Transfer learning was effective when downsampling to just 500 bound/unbound regions (Fig. 4 for HNF4A; Figs. S2, S3, S4 and S5 for the remaining TFs), which was concordant with the previous result for ATF4. For JUND and MAX, transfer learning was effective when downsampling to as few as 50 bound/unbound regions (Figs. S2F and S3F). This could be explained by the use of data for both TFs in the pre-training step. However, for SP1, which had also been used to train the multi-model, transfer learning was no longer effective when downsampling below 500 bound/unbound regions (Fig. S5C). We attributed this result to the poor performance of SP1 in the multi-model (AUCPR = 0.796 vs. 0.882 and 0.866 for JUND and MAX, respectively; Table S2), suggesting that the multi-model could not correctly learn the binding features of SP1, which in turn would result in an inadequate set of weights with which to initialize the individual model in the fine-tuning step.

Finally, since the TF binding matrix aggregated data across 52 cell and tissue types, we wondered if that would have an impact on transfer learning. We focused on GM12878 and K562 cells, which covered the largest number of TFs in the matrix. Using resolved regions in both GM12878 and K562 cells, we pre-trained a multi-model for 50 TFs common to both cell types, but only using matrix values from GM12878 cells (i.e., the multi-model was not trained using data from K562 cells). GM12878-specific resolved regions (i.e., regions resolved in GM12878 but not in K562 cells) and K562-specific resolved regions were held-out for fine-tuning. Using the held-out data, we then trained individual models, with and without transfer learning, for 35 of the 50 TFs used in the pre-training step (15 TFs were discarded because they had < 250 resolved regions left non-overlapping with multi-model regions). We additionally trained individual models (with and without transfer learning) for 76 TFs with resolved regions in either GM12878 cells (2), K562 cells (55), or both (19). Pre-training and fine-tuning on the same or on different cells did not impact transfer learning (Welch *t* test, *p* value = 0.202, NS; Fig. S6A); however, individual models pre-trained and fine-tuned in the same cells benefited from transfer learning significantly more (Welch *t* test, *p* value = 0.029; Fig. S6B), although differences were small (0.166 vs. 0.134). We attributed this to the poorer performance of individual models trained from scratch on GM12878 cells compared to those trained on K562 cells for the 35 multi-model TFs (average AUCPR = 0.656 vs. 0.713; Wilcoxon signed-rank test, *p* value = 0.001; Fig. S6D). Taken together, these results suggest that aggregating the data in a cell and tissue type-agnostic manner did not introduce any bias during the construction of the TF binding matrix.

### Biologically relevant prior knowledge improves transfer learning

**Fig. 5:**
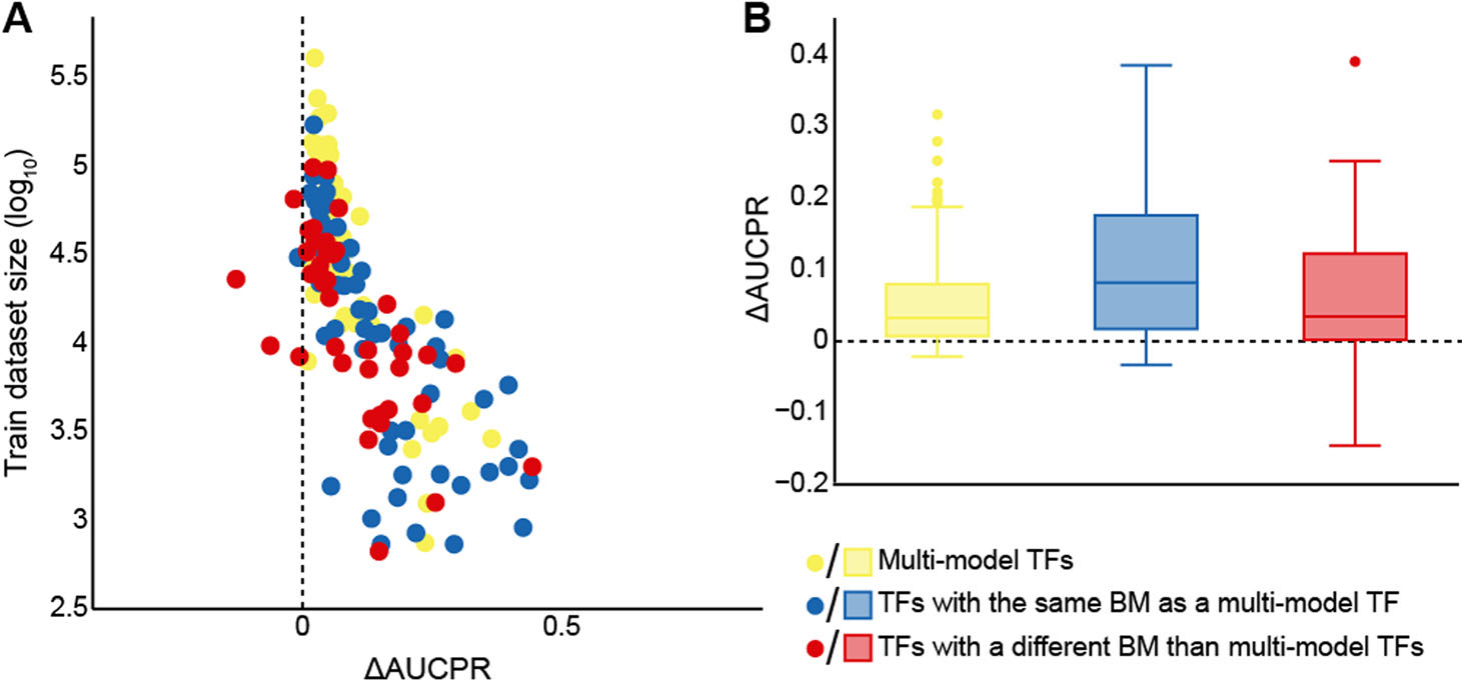
Transfer learning is more beneficial for TFs with the same binding mode as a TF from the pre-training step. **A** Performance difference (i.e., ΔAUCPR) of individual models trained with and without transfer learning (*x*-axis) is plotted with respect to the size of the training dataset (*y*-axis) for TFs used to train the multi-model (yellow dots), TFs with the same binding mode as a multi-model TF (blue dots), and TFs with different binding modes than multi-model TFs (red dots). **B** ΔAUCPRs for TFs used to train the multi-model (yellow boxes), TFs with the same binding mode as a multi-model TF (blue boxes), and TFs with different binding modes than multimodel TFs (red boxes). AUCPR, area under the precision-recall curve; ΔAUCPR, AUCPR from transfer learning - AUCPR from training from scratch; BM, binding mode; TF, transcription factor

TFs from the same family often have highly similar DNA-binding specificities [44]; hence, we hypothesized that the presence in the pre-training step of TFs with the same binding mode as the target TF could have a positive effect on transfer learning performance. The JASPAR database of TF binding profiles includes a hierarchical clustering that groups TFs based on the similarity of their DNA-binding profiles [45]. We relied on this grouping as the source of binding modes (Table S3). Focusing on the set of TFs whose performance worsened with transfer learning, we found that the binding modes of the most extreme cases (ΔAUCPR ≥ 0.025), namely MEF2A, MEF2C, HOXB13, TBX21, and TFAP2A, differed from those of the 50 TFs used to train the multi-model, suggesting that the inclusion in the pre-training step of TFs with relevant binding modes could be beneficial. This observation was further supported by reviewing the remaining TFs; TFs with the same binding mode as a TF from the pre-training step benefited from transfer learning significantly more (Welch *t* test, *p* value = 0.035; Fig. 5).

**Fig. 6:**
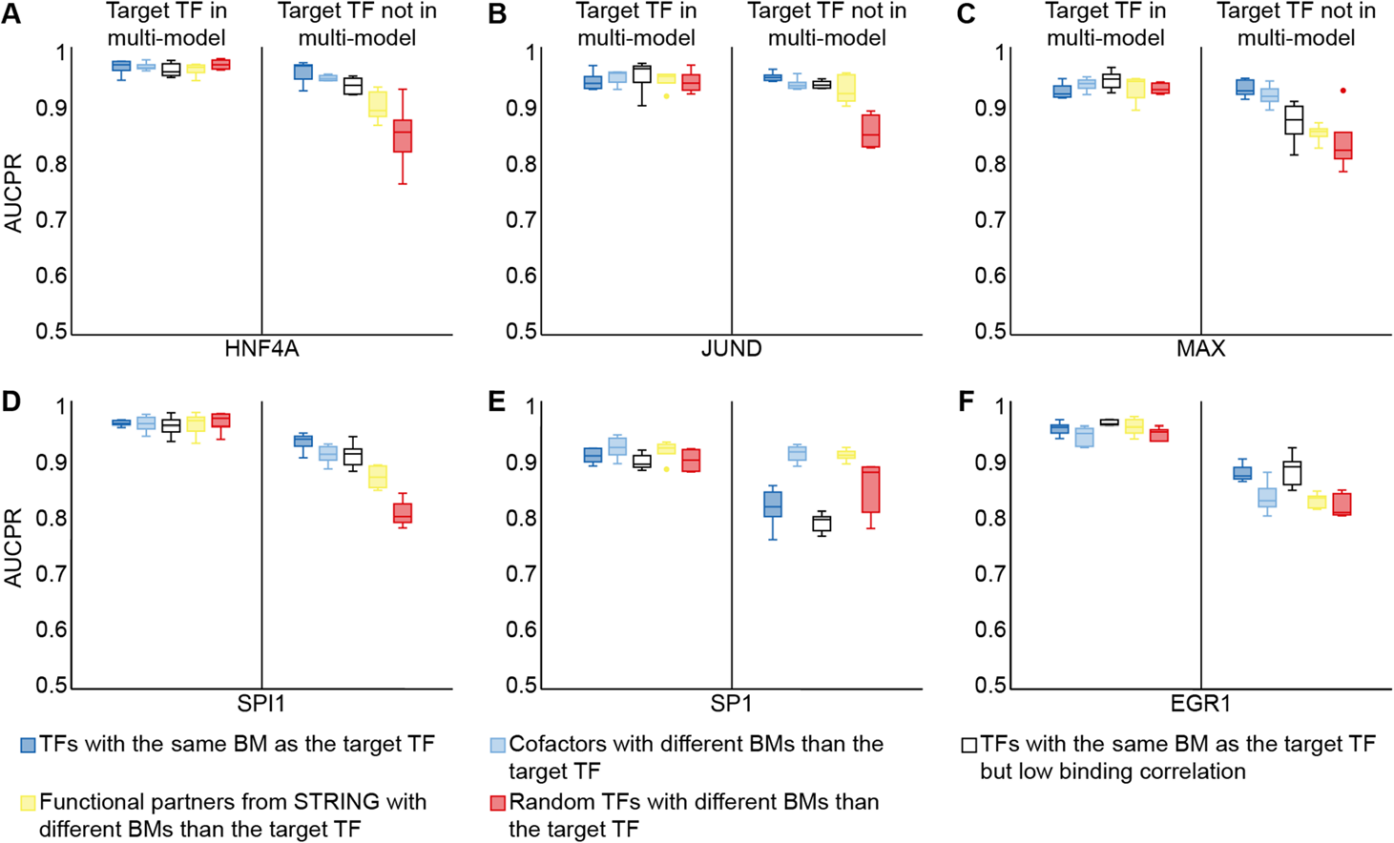
Biologically relevant transfer learning improves model performance. Transfer learning performance for the target TFs HNF4A (**A**), JUND (**B**), MAX (**C**), SPI1 (**D**), SP1 (**E**), and EGR1 (**F**), from multi-models pre-trained with five TFs with the same binding mode as the target TF (dark blue boxes), five cofactors of the target TF with a different binding mode than the target TF (light blue boxes), five non-cofactors with the same binding mode as the target TF (white boxes), five functional partners of the target TF from STRING with a different binding mode than the target TF (yellow boxes), and five randomly selected TFs with a different binding mode than the target TF (red boxes), with (left) and without (right) the presence of the target TF in the pre-training step. AUCPR, area under the precision-recall curve; BM, binding mode; TF, transcription factor

We hypothesized that the presence in the pre-training step of other biologically relevant prior knowledge, such as cofactors, or functional partners from STRING, could also have a positive effect on transfer learning performance (Methods). We defined cofactors as pairs of TFs whose binding was positively correlated. We focused on the same pentad of TFs from the previous section (i.e., HNFA4, JUND, MAX, SPI1, and SP1). For each TF, we pre-trained five different multi-models with five TFs with the same binding mode as the target TF, five cofactors of the target TF, five TFs with the same binding mode as the target TF but whose binding was not correlated with it (i.e., non-cofactors), five functional partners of the target TF from STRING, and five randomly selected TFs. Cofactors, functional partners from STRING, and randomly selected TFs were restricted to have different binding modes than the target TF. To avoid any confounding effects related to the training dataset size, all models were trained with a similar number of regions: ~ 70,000 for multi-models and ~ 2000 for individual models. Furthermore, to set an upper performance limit for each pre-training strategy, we repeated the analysis by replacing one of the five TFs, with which we pre-trained the multi-model, by the target TF. As expected, pre-training with the target TF resulted in better transfer learning. Moreover, when the target TF was included in the multi-model, we did not observe any significant differences between the five pre-training strategies (Kruskal-Wallis *H* test, Bonferroni adjusted *p* values = NS; Fig. 6). In contrast, when the target TF was not included in the multi-model, pre-training with either TFs with the same binding mode as the target TF or with cofactors were the best strategies: both achieved effective performance levels for four out of five TFs (except for SP1). Interestingly, using non-cofactors with the same binding mode as the target TF during pre-training led to slightly worse performance, suggesting that cofactors could play an important role in TF binding prediction. Of the five TFs analyzed, SP1 was a notable outlier: not only did it show the worst performance levels overall, but when it was not included in the multi-model, pre-training with other Krüppel-like zinc fingers sharing the same binding mode as SP1 performed worse than pre-training with randomly selected TFs (Fig. 6E). To confirm whether these observations were general to this family of TFs, we repeated the experiment with another Krüppel-like zinc finger, EGR1. We obtained results more in line with the other four TFs (Fig. 6F), suggesting that SP1 was an isolated case. Finally, to ensure that these results were not biased by our choice of model architecture, we repeated the previous experiment using the hybrid architecture with convolutional and recurrent layers of DanQ [46], obtaining similar results (Fig. S7).

**Fig. 7:**
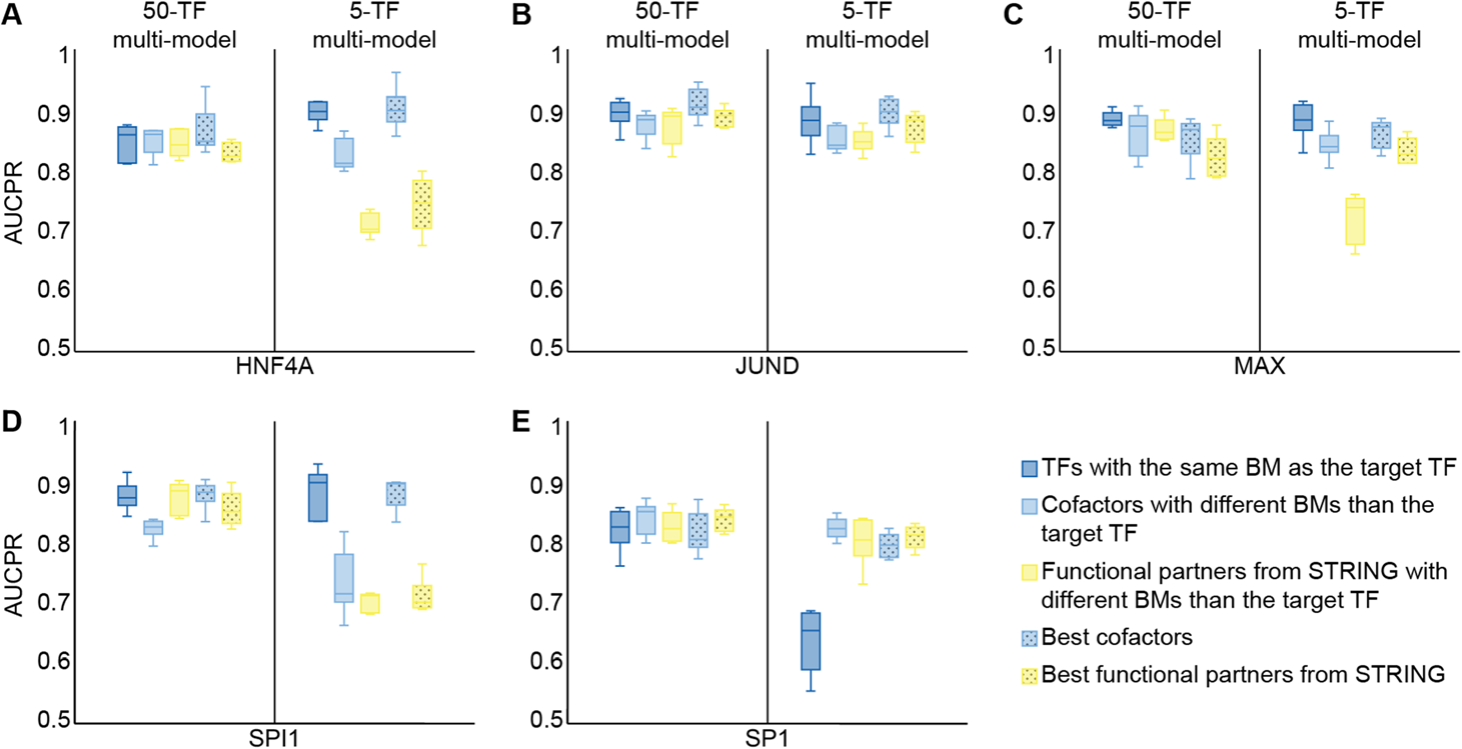
Pre-training with a few biologically relevant TFs only offers a slight advantage over a multi-model pre-trained with a large number of TFs. Transfer learning performance for the target TFs HNF4A (**A**), JUND (**B**), MAX (**C**), SPI1 (**D**), and SP1 (**E**): on the right, from multi-models pre-trained with five TFs with the same binding mode as the target TF (dark blue boxes), five cofactors of the target TF with a different binding mode than the target TF (light blue boxes), five functional partners of the target TF from STRING with a different binding mode than the target TF (yellow boxes), and the best cofactors and functional partners from STRING of the target TF regardless of their binding mode (dotted light blue and yellow boxes, respectively); on the left, from “burying” each group of five biologically relevant TFs among 45 TFs drawn from the original multi-model, each with a different binding mode than the target TF. The performance of de novo PWMs (black stars) is provided as a baseline for each TF. AUCPR, area under the precision-recall curve; BM, binding mode; PWM, position weight matrix; TF, transcription factor

Next, we tested whether focusing on a few biologically relevant TFs, rather than pre-training with a large dataset, would be a more effective transfer learning strategy. For each category of biologically relevant information (i.e., binding modes, cofactors and functional partners from STRING), we pre-trained two different multi-models for each of the pentad TFs with either five biologically relevant TFs, or “burying” the same five TFs in 45 TFs drawn from the original multi-model, each with a different binding mode than the target TF. For this set of experiments, the target TF was not included in the pre-training step, and we fixed the training dataset sizes of multi-models and individual models to ~ 40,000 and ~ 2000 regions, respectively. Furthermore, we used cofactors and functional partners from STRING with and without sharing the same binding mode as the target TF. We observed that pre-training with just five biologically relevant TFs led to only slightly better performance levels (Fig. 7).

**Fig. 8:**
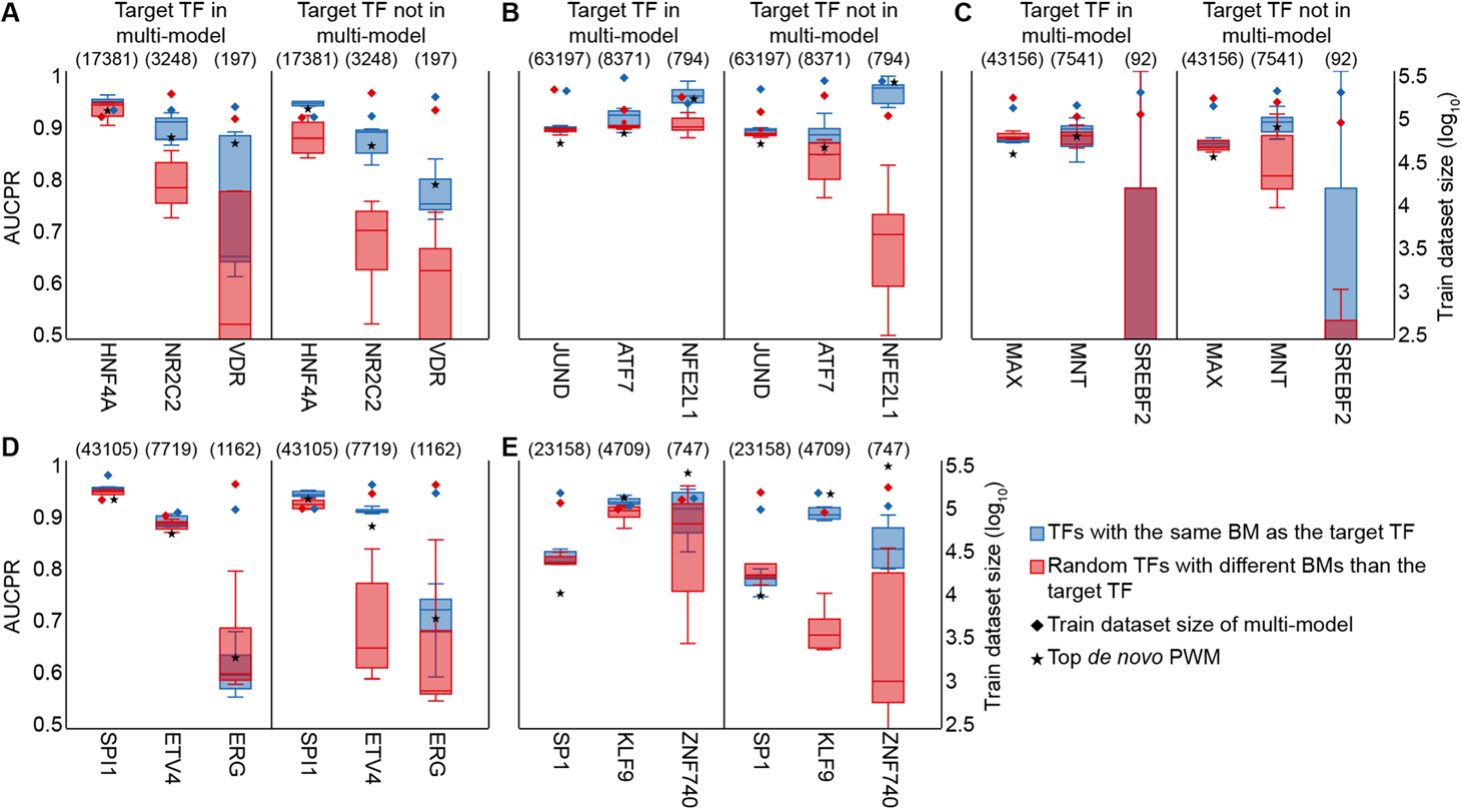
The use of relevant binding mode information in the pre-training step improves model performance for TFs with small training datasets, regardless of their presence in the multi-model. Transfer learning performance for three target TFs with the same binding mode, but different number of bound regions, from each of the following five families: **A** HNF4A, NR2C2, and VD2R (nuclear receptors); **B** JUND, ATF7, and NFE2L1 (basic leucine zippers); **C** MAX, MNT, and SREBF2 (basic helix-loop-helix factors); **D** SPI1, ETV4, and ERG (tryptophan cluster factors); and **E** SP1, KLF9, and ZNF740 (C2H2 zinc fingers). For transfer learning, multi-models were pre-trained with five TFs with the same binding mode as the target TF (dark blue boxes), or five randomly selected TFs with a different binding mode than the target TF (red boxes), with (left) and without (right) the presence of the target TF in the pre-training step. The training dataset size of each multi-model is indicated with diamonds (secondary *y*-axis). The number of bound regions for each TF is shown between parenthesis. AUCPR, area under the precision-recall curve; BM, binding mode; TF, transcription factor

The pentad TFs had a sufficiently large number of bound regions that we downsampled; hence, we wondered whether TFs with less bound regions would behave similarly. For each of the pentad TFs, we selected two additional TFs with the same binding mode and within the following ranges of bound regions: < 1000 positives or 1000–10,000 positives. For each of these 15 TFs (the five “pentad” TFs plus the 10 newly selected), we trained individual models using transfer learning from two different multi-models pre-trained with either five TFs with the same binding mode as the target TF, or five randomly selected TFs with different binding modes than the target TF. Each multi-model was trained both with and without the target TF included in the pre-training step. Unlike the above set of experiments, this time, we did not perform downsampling. In general, pre-training with the target TF resulted in better transfer learning performance (Fig. 8); however, for TFs with large datasets, this effect was less obvious. In addition, pre-training with a biologically relevant multi-model improved transfer learning performance, even when the target TF was present in the multi-model, especially for TFs with < 10,000 positives. Similar results were obtained when the experiment was repeated, but using longer input sequences (Figs. S8 and S9), as well as pre-training using five cofactors (Fig. S10). Finally, except for KLF9 and ZNF740, both zinc fingers, and SREBF2, which only had ~ 41.2 training and ~ 5.4 test sequences per data split (and hence should be considered an outlier), biologically relevant transfer learning performed similar than or better to de novo PWMs (Figs. 8 and S8, S9 and S10).

### Interpretation of the transfer learning mechanism

**Fig. 9:**
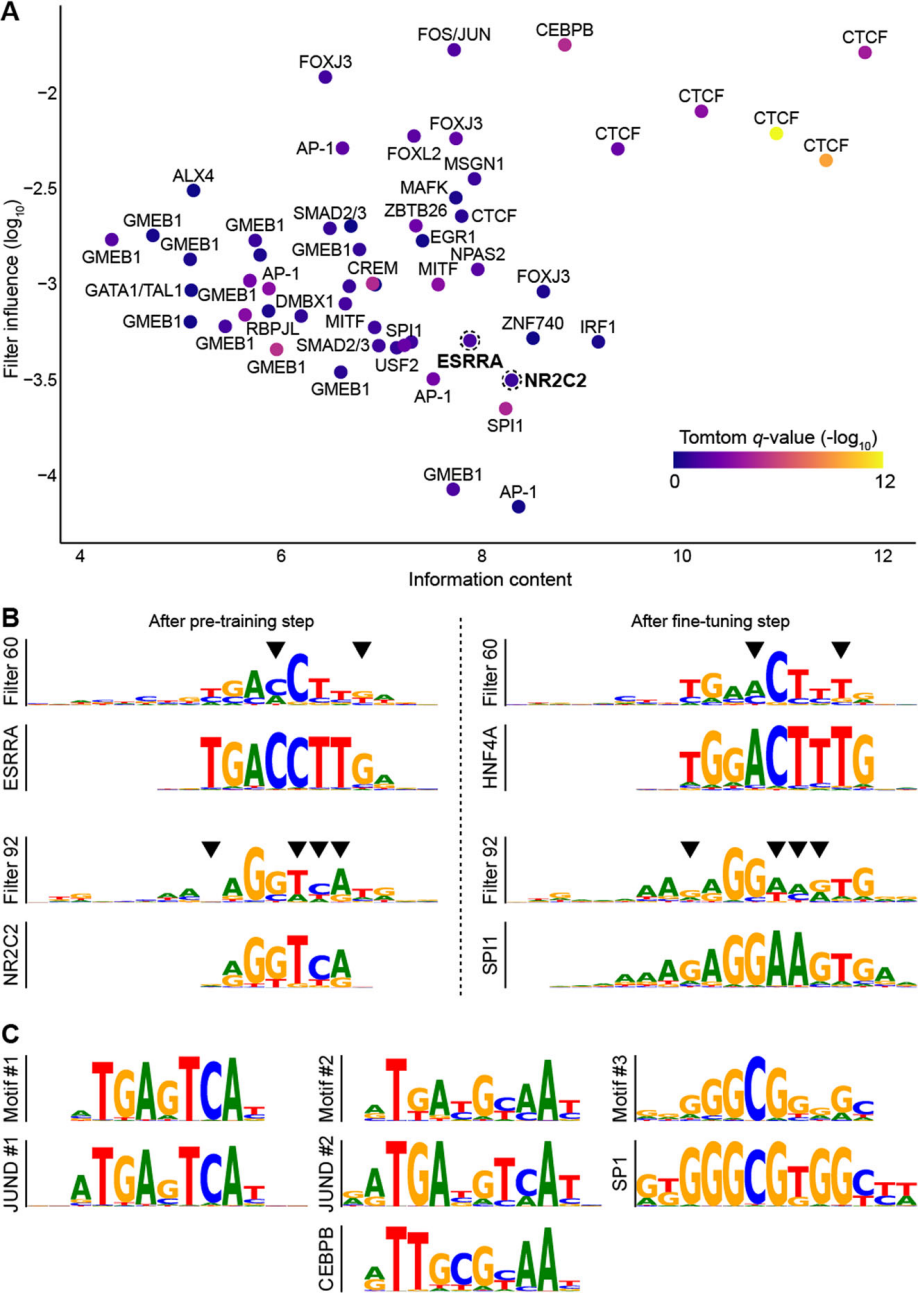
Model interpretation elucidates the transfer learning mechanism. **A** TF motif representations learnt in the first convolutional layer of the original multi-model of 50 TFs. Filters (dots) are plotted with respect to their information content (*x*-axis) and overall influence (*y*-axis). The color-scale reflects the Tomtom similarity of a given filter to a TF profile from the JASPAR database. When applicable, the name of the most significant TF is shown. Filters 60 and 92 correspond to the JASPAR profiles for ESRRA and NR2C2 (shown in bold), respectively, and are highlighted with dotted line circles. **B** Transfer learning fine-tunes the filters learned in the pre-training step to resemble the binding motif of the target TF. Specific positions (black triangles) from filters 60 and 92 (highlighted above) are refined in the fine-tuning step to resemble the JASPAR profiles for HNF4A and SPI1, respectively. **C** Learning the motifs of cofactors contributes to the predictive capacity of the model. Using DeepLIFT importance scores from a JUND model initialized with the weights of a multi-model pre-trained with five cofactors, TF-MoDISco recovers the motifs of two of the cofactors, CEBPB and SP1, as well as the JUND motif

In an attempt to understand the mechanism of transfer learning, we converted the filters from the first convolutional layer of the original multi-model to PWMs and compared them using Tomtom [47] to TF binding profiles from JASPAR (Methods; Fig. 9A). As expected, the majority of filters (55%) had significant similarities to known JASPAR profiles (Tomtom *q* value ≤ 0.05; Table S4). Focusing on HNF4A, which had not been used in the pre-training step, we found that four of the multi-model filters were refined in the fine-tuning step to resemble the motifs of HNF4A (Fig. 9B). We also observed that an increased number (six) of filters became similar to the motifs of HNF4A after using transfer learning compared to training from scratch (three; Table S4). Moreover, using transfer learning, the individual model of HNF4A was able to learn the two distinct binding modes of HNF4A represented by the JASPAR profiles MA0114.4 and MA1494.1. Similar observations were made for SPI1, which was also not present in the pre-training step (Fig. 9B; Table S4). Taken together, these findings suggest that weights from the pre-training step provided a better initialization for convolutional filters in the fine-tuning step, wherein they were refined to resemble the binding motif of the target TF. This would, in turn, explain the increased performance of transfer learning compared to training from scratch. To confirm that the refinement of convolutional filters in the fine-tuning step was responsible for the improvement in model performance by transfer learning, we applied an alternative fine-tuning strategy: we froze the convolutional layers of the pre-trained multi-model and trained only on the fully connected layers (i.e., no refinement of convolutional layer filters was allowed). Doing so resulted in poorer model performance, particularly for TFs that were not present in the multi-model (Table S5), further supporting the importance of filter refinement in the fine-tuning step.

Next, we applied DeepLIFT [48] and TF-MoDISco [49] in an attempt to understand the role of cofactors in transfer learning. Briefly, DeepLIFT quantifies the importance to the model prediction of each nucleotide in a given genomic sequence, while TF-MoDISco clusters “important” nucleotides from different genomic sequences into motifs. We show an analysis of transfer learning for JUND from two different multi-models pre-trained on either five cofactors with a different binding mode than JUND (CEBPB, MAFF, MAFG, NFIC, and SP1) or five TFs from five different families whose binding was not correlated with JUND (CTCF, EBF1, MXI1, NFYA, and TCF3). Data for JUND was not present in the pre-training step. The three motifs generated by TF-MoDISco for the cofactor model corresponded to the canonical JASPAR profiles of CEBPB and SP1, both of which were among the cofactors used to train the multi-model, as well as JUND (Fig. 9C). For the second model trained on five unrelated TFs, TF-MoDISco identified five motifs, none of which corresponded to the canonical JUND motif. Taken together, these results suggest that the model used the presence of CEBPB and SP1 motifs to aid in the prediction of JUND binding.

Finally, to further confirm the central role of convolutional layers, particularly filters, in the transfer learning process, we focused on the 10 TFs from Fig. 8 with an intermediate (1000–10,000) and small (< 1000) number of bound regions. We trained individual models with transfer learning wherein we transferred the weights of either the first or all three convolutional layers (i.e., we did not transfer the weights from the fully connected layers). Doing so only slightly worsened performance compared to the original transfer learning strategy, particularly when we transferred all three convolutional layers (Fig. S11). We also explored the importance of convolutional filters by initializing individual models with a combination of non-redundant profiles from the JASPAR database and de novo PWMs (Methods). As previously reported [46], filter initialization with JASPAR profiles, and in our case de novo PWMs, improved model performance compared to training from scratch; however, performance was inferior to that of transfer learning (Fig. S11).

## Discussion

In this study, we have demonstrated how incorporating prior knowledge via transfer learning can improve TF binding prediction in deep learning methods, with notable benefits for TFs with scarce experimental binding data. We constructed a large data matrix of TF binding events through the combination of DNA accessibility with experimental and computational TF binding information to define TF-bound and unbound regions with high confidence. Using this matrix, we implemented a two-step transfer learning approach for TF binding prediction that first draws data from a large number of TFs to learn the general properties of regulatory regions and, second, exploits these properties to generate a specific deep learning model for a single TF. We introduced methods that allowed us to study the learned properties in the deep learning models, providing insight into the transfer learning mechanism. We confirm not only the benefits of including binding data from homologs of the target TF into the first step of transfer learning (consistent with studies predating deep learning [50]), but also the importance of including cofactors. Finally, when focusing specifically on TFs for which experimental binding data is scarce, we show that transfer learning is routinely successful in generating a deep learning model from only 500 experimental regions, and in a few extreme cases, it can be successful from as few as 50 regions.

While other studies have reported similar findings about the benefits of transfer learning for TF binding prediction [23, 24], we demonstrate that the effectiveness of transfer learning depends largely on the functional association between the target TF and those included in the pre-training step. We show improvements when pre-training with TFs that have the same binding mode as the target TF compared to pre-training with randomly selected TFs. Similar benefits are observed for other biological categories such as cofactors or, to a lesser extent, functional partners from STRING. However, for TFs with large datasets, if the target TF is included in the multi-model, then transfer learning performance is similar for biologically relevant and randomly selected pre-training sets of TFs. For TFs with small training datasets, there is a greater difference (i.e., transfer learning performance is higher) between pre-training with biologically relevant or randomly selected TFs, even when the target TF is included in the multi-model. One possible explanation is that if a TF has enough training examples, then the model can infer its binding without any additional aid (from cofactors or binding mode information). In contrast, when the target TF has few bound regions (relative to other TFs), their inclusion in the multi-model stage will result in the inability of the model to learn relevant features of the target TF for subsequent fine-tuning. In this situation, the inclusion of biologically relevant information (e.g., cofactors) may be of benefit.

The idea of using biologically relevant prior knowledge of the target TF in the pre-training step is enticing; however, it can be challenging to do so: training a separate multi-model for each TF is computationally expensive. Furthermore, obtaining prior knowledge for a specific TF could prove difficult. For instance, it may not be straightforward to identify cofactors for a TF with few ChIP-seq peaks, as its binding vector may not correlate well with those of other TFs. Binding mode information may be unavailable for new TFs or for TFs from families with fewer members. Lastly, the STRING data available for some TFs could be of low confidence. With these limitations, one might consider pre-training a larger multi-model with most, if not all, of the existing binding modes. Such a multi-model would be pre-trained with the best representative TF (i.e., the TF with the largest training dataset) from each binding mode, would be more generalizable to other TFs, and would avoid having to pre-train a separate multi-model for each specific case. While our findings, particularly those from the experiment wherein we “buried” five biologically relevant TFs in 45 additional TFs, show that in most cases pre-training on a few biologically relevant TFs results in better transfer learning, the generalized approach with 50 TFs contributing to the multi-model performed nearly as well. Optimizing the size and properties of the pre-training dataset to maximize binding mode diversity is likely to be a fruitful avenue of future research.

For image recognition, deep learning models tend to learn simple and basic features in the first layers (lines, curves, etc.), whereas more complex and resolved features emerge at deeper layers. Therefore, it is commonplace to train new models by taking big pre-trained models (multi-models in our case) and freezing the initial layers, focusing the fine-tuning onto the deeper layers. We demonstrate that such an approach does not work for TF binding, as it is likely that the main features, such as TF binding motifs, are already learned in the first convolutional layers.

We have not explored the impact of the learning rate on transfer learning performance. Often, a small learning rate is applied when one uses a pre-trained model based on a CNN. However, an extremely low learning rate makes it challenging for the model to learn new features. In contrast, with high learning rates, weights from the pre-trained model can be ignored, resulting in the loss of the prior knowledge. In this study, when fine-tuning individual models, we used a learning rate 10 times lower than that used to train multi-models. Further exploration of the impact of learning rate on resolving motifs from the data may be a focus of future studies. As for the learning rate, the batch size parameter should also be explored as recent studies have highlighted its impact on model performance and transfer learning efficacy [51, 52].

Our work demonstrates that transfer learning improves TF binding prediction, particularly when data is limited. As our goal was neither performance optimization relative to other methods nor computational efficiency, there remains opportunity for innovation in transfer learning strategies for both TF binding prediction and, more broadly, for motif analysis across bioinformatics.

## Conclusions

Existing deep learning frameworks readily allow the transfer of learned properties between models within the same architecture, overcoming the computational cost and reducing the amount of training data required. This study is a demonstration of one approach to improving the performance of deep learning models for TF binding prediction via transfer learning through the incorporation of field-/domain-specific information. Our results advocate for a broader adoption of such focused transfer learning strategies in deep learning, particularly for biological sequence analysis.

## Methods

### TF binding matrices

We built two TF binding matrices (i.e., data structures containing information about TF binding events, not motif models), one more sparse for training individual models and the other (less sparse) suitable for training multi-models. The matrices aggregate the binding data of 163 TFs for 1,817,918 accessible genomic regions across 52 cell and tissue types, with rows representing TFs and columns representing accessible genomic regions (Fig. 1). As the source of TF binding events, we used ChIP-seq peak summits from ReMap 2018 [7] and PWM-based TFBS predictions from UniBind [10]. We used the track of ENCODE DHS peak clusters from the UCSC Genome Browser [53, 54] as the accessible genomic regions. Regions were resized, from an average length of ~ 217 bp to 200 bp around the center of each DHS cluster using bedtools slop [55]. Data was matched by cell and tissue type. For the sparse matrix (i.e., for training individual models), each element (i.e., a TF-DHS pair) was assigned one of three values: “1” (i.e., bound) if the region was accessible (i.e., DHS positive) and overlapped with both ReMap (i.e., ChIP-seq peak positive) and UniBind (i.e., TFBS positive) in at least one matched cell or tissue type, “0” (i.e., unbound) if the region was accessible but did not overlap with both binding features of the TF in any matched cell and tissue type, or “null” if the binding of the TF to the region could not be resolved. A null value indicated insufficient evidence to determine if the region was or was not bound by the TF: the region may not be accessible (i.e., DHS negative) in any matched cell or tissue type for which the TF binding had been profiled, or it may be accessible but only overlap with one type of binding feature of the TF (i.e., ReMap-peak or UniBind motif, but not both). For the less sparse matrix (i.e., for training multi-models), unresolved elements (i.e., null), wherein the region was accessible but only overlapped with one type of binding feature of the TF (not both), were instead assigned a value of 0 (i.e., unbound) to make it suitable for size reduction when training multi-models.

### Cosine similarity

The binding vector of a TF was given by the row in the sparse matrix corresponding to that TF. For a pair of TFs, the cosine similarity between their binding vectors was computed using scikit-learn [56]. Unresolved regions of either TF were removed from both vectors prior to calculation.

### Model architecture

We adapted the CNN architecture from Basset [57] and AI-TAC [58] for TF binding prediction:

- 1st convolutional layer with 100 filters (19 × 4), batch normalization, ReLU activation, 0% dropout, and max pooling (3 × 3);
- 2nd convolutional layer with 200 filters (7 × 1), batch normalization, ReLU activation, 0% dropout, and max pooling (3 × 3);
- 3rd convolutional layer with 200 filters (4 × 1), batch normalization, ReLU activation, 0% dropout, and max pooling (3 × 3);
- 1st fully connected layer with 1000 nodes, batch normalization, ReLU activation, and 30% dropout;
- 2nd fully connected layer with 1000 nodes, batch normalization, ReLU activation, and 30% dropout; and
- Fully connected output layer with 1, 5, or 50 outputs (depending on the model).

For DanQ [46], we used the following specifications:

- 1st convolutional layer with 320 filters (26 × 4), ReLU activation, 20% dropout, and max pooling (13 × 13);
- 2 bi-directional LSTM layers with hidden state size 320 and 50% dropout;
- 1st fully connected layer with 925 nodes and ReLU activation; and
- Fully connected output layer with 1, 5, or 50 outputs (depending on the model).

Both architectures were implemented using the PyTorch framework [59].

### Transfer learning

We implemented a two-step transfer learning strategy similar to that used in AgentBind [24]. In the pre-training step, we trained a multi-model. In the fine-tuning step, we initialized the model of the target TF by transferring all of the layers learned by the multi-model, except the output layer. We then trained the initialized model of the target TF with a 10-fold lower learning rate (i.e., 0.0003). Unless otherwise specified, in the fine-tuning step, we trained the entire model including the convolutional and fully connected layers.

To train multi-models, we used the less sparse matrix. We extracted a slice of the matrix containing the row vectors of all TFs included in the multi-model. Any column vectors containing unresolved elements were removed. Then, we randomly split the regions into training (80%), validation (10%), and test (10%) sets using scikit-learn. The training and validation sets additionally included the reverse-complement of each region. We trained the model using the Adam optimizer [60]. We applied one-hot encoding to convert nucleotides into 4-element vectors (i.e., A, C, G, and T), set the learning rate to 0.003 and batch size to 100, and used an early stopping criteria to avoid overfitting when the model performance on the validation set did not improve. Sequences with Ns were discarded.

Individual models were trained in a similar way, but using the sparse matrix. The number of bound versus unbound regions for all TFs was imbalanced. For example, one of the most abundant TFs in the matrix, CTCF, had 1,656,242 resolved regions of which < 5% were bound. To deal with the imbalance, we downsampled the set of unbound regions to a 50:50 ratio while accounting for the %GC content distributions between bound and unbound regions. To ensure the robustness of our results, each individual model was trained five times with different, randomly generated training/validation/test splits. To avoid overfitting, there was no overlap between the regions used in the pre-training and fine-tuning steps within the same experiment.

### Model performance

Model performance was evaluated using the area under the precision-recall (AUCPR) curve calculated with scikit-learn. To calculate the performance of a TF in the multi-model (Fig. 3B), we used the predictions on the test sequences by the output node in the multi-model corresponding to that TF.

### Pre-training with biologically relevant TFs

The pentad TFs used in the analyses (i.e., HNFA4, JUND, MAX, SP1, and SPI1) belonged to the following binding modes: 2 and 4 (HNF4A), 1 and 18 (JUND), 7 (MAX), 34 (SP1), and 16 (SPI1). At least five additional TFs in the sparse matrix shared one or more binding modes with a pentad TF. For multi-models trained with the target TF, we randomly selected four other TFs sharing one or more binding modes with the target TF (five when training without the target TF).

To identify potential cofactors based on correlated binding, we computed pairwise cosine similarities between the binding vectors of 162 TFs (SMAD3 was removed because it did not have any bound regions in the sparse matrix). For a given TF, we sorted the remaining 161 TFs by cosine similarity and removed those sharing one or more binding modes with it. Out of the remaining TFs, we selected the top five for multi-model training. If the multi-model was trained with the target TF, we only selected the top four TFs. When focusing on the best cofactors in Fig. 7, we kept all TFs after sorting. Similarly, to select TFs with low binding correlation that shared one or more binding modes with the target TF, we removed those with different binding modes than the target TF after sorting the TFs by cosine similarity and selected the bottom five TFs. Again, when training the multi-model with the target TF, only the bottom four TFs were selected.

The STRING database stores known and predicted protein-protein interactions, and provides a confidence score for the interactions; we used version 11.0 [42]. We defined the functional partners of a TF as its set of interactors from STRING. To pre-train with prior knowledge from STRING-based associations, we sorted the functional partners of the target TF by confidence score and removed those that shared one or more binding modes with it. The top five TFs were selected for pre-training (the top four if pre-training with the target TF). As with selecting the best cofactors, when focusing on the best functional partners from STRING in Fig. 7, we kept all TFs.

Finally, to pre-train on random TFs, we randomly selected five TFs (or four if pre-training with the target TF) with different binding modes than the target TF.

### Model interpretation

To interpret the performance of the original multi-model, we converted each of the 100 filters from the first convolutional layer to PWMs, as in AI-TAC. For each filter, we constructed a position frequency matrix (PFM) from all 19-mers (i.e., DNA sequences of length 19) that activated that filter by ≥ 50% of its maximum activation value in all correctly predicted regions. PFMs were then converted to PWMs using scripts from [58], and the background uniform nucleotide frequency was set to 0.25. The resulting PWMs were compared to vertebrate profiles from the JASPAR 2020 database using Tomtom (version 5.0.5) [47].

The influence of each filter in the multi-model was obtained by “silencing” that filter and computing the impact on the model’s predictive capacity. Silencing was achieved by setting the activation values of the filter across all samples in the batch to zero. The resulting output was passed through the remaining layers of the model to obtain the prediction values after silencing. Using this approach, we computed the average influence value for each filter in the model by averaging the square of the differences between the actual and silenced predictions.

We generated DeepLIFT [48] importance scores with 10 reference sequences for each positively predicted sample in the test set using the Captum library [61]. To obtain motifs from DeepLIFT importance scores, we used TF-MoDISco [49] with default settings.

### De novo PWMs

For each TF and for each data split, we generated five de novo PWMs by applying STREME (version 5.3.0) [62] on the set of training sequences. We set the fraction of sequences held-out for *p* value computation to 0 (option --hofract), the maximum PWM length to 21 bp (i.e., the length of CTCF profile as reported in JASPAR, which was the longest in our dataset; option --maxw), and the number of output PWMs to 5 (option --nmotifs). We then computed sum occupancy scores (i.e., the sum of probabilities obtained from sliding the PWM along the forward and reverse complementary strands of a sequence [44]) on the set of test sequences using PWMScan (version 1.1.9) [63]. Performance of the top de novo PWM (i.e., motif 1 in the STREME output) was evaluated by means of AUCPR.

### Initialization with JASPAR profiles and de novo PWMs

Filter weights of individual models were initialized with 50 non-redundant JASPAR profiles (Table S6), as well as 10 de novo PWMs (i.e., the five de novo PWMs from STREME in the forward and reverse complementary orientations); the remaining filters were initialized normally. To obtain the set of 50 non-redundant JASPAR profiles, we started by randomly selecting one profile per binding mode, for a total of 111 profiles. We then computed pairwise similarities between the 111 profiles in the forward and reverse complementary orientations using Tomtom and randomly selected 50 dissimilar profiles (i.e., all vs. all Tomtom *q* values > 0.05). PWMs were converted to filter weights following specifications from the DanQ manuscript: We resized PWMs to 19 bp and then subtracted 0.25 from the probability of each nucleotide at each position. Previously, JASPAR profiles were reformatted to PWMs using Biopython [64].

## Supplementary Information

**Additional file 1: Fig. S1.** Performance variance (i.e., σ; y-axis) by means of AUCPR of individual models trained with (blue) and without transfer learning (red) is plotted with respect to the size of the training dataset (x-axis) for 148 TFs. AUCPR = area under the precision-recall curve; TF = transcription factor; TL = transfer learning.

**Additional file 2: Fig. S2.** Performance of JUND models trained with (blue boxes) and without (red boxes) transfer learning on 5,000 (**A**), 1,000 (**B**), 500 (**C**), 250 (**D**), 125 (**E**), and 50 (**F**), bound and unbound regions. Each model was trained five times with different random initializations to ensure the robustness of the results. AUCPR = area under the precision-recall curve; TF = transcription factor; TL = transfer learning.

**Additional file 3: Fig. S3.** Performance of MAX models trained with (blue boxes) and without (red boxes) transfer learning on 5,000 (**A**), 1,000 (**B**), 500 (**C**), 250 (**D**), 125 (**E**), and 50 (**F**), bound and unbound regions. Each model was trained five times with different random initializations to ensure the robustness of the results. AUCPR = area under the precision-recall curve; TF = transcription factor; TL = transfer learning.

**Additional file 4: Fig. S4.** Performance of SPI1 models trained with (blue boxes) and without (red boxes) transfer learning on 5,000 (**A**), 1,000 (**B**), 500 (**C**), 250 (**D**), 125 (**E**), and 50 (**F**), bound and unbound regions. Each model was trained five times with different random initializations to ensure the robustness of the results. AUCPR = area under the precision-recall curve; TF = transcription factor; TL = transfer learning.

**Additional file 5: Fig. S5.** Performance of SP1 models trained with (blue boxes) and without (red boxes) transfer learning on 5,000 (**A**), 1,000 (**B**), 500 (**C**), 250 (**D**), 125 (**E**), and 50 (**F**), bound and unbound regions. Each model was trained five times with different random initializations to ensure the robustness of the results. AUCPR = area under the precision-recall curve; TF = transcription factor; TL = transfer learning.

**Additional file 6: Fig. S6.** (**A**) Performance of transfer learning models trained on either GM12878 (blue boxes) or K562 cell data (red boxes). (**B**) Performance difference (i.e., ΔAUCPR) of individual models trained with and without transfer learning on either GM12878 or K562 cell data. Performance of individual models trained with (**C**) and without (**D**) transfer learning on either GM12878 or K562 cell data for 35 multi-model TFs, as well as for 76 additional TFs with resolved regions in either GM12878 cells (2), K562 cells (55), or both (19; i.e., GM12878/K562 TFs). (**E**) Performance difference of individual models trained with and without transfer learning on GM12878 or K562 cell data for the previous TF categories. Transfer learning models were pre-trained using data from GM12878 cells. AUCPR = area under the precision-recall curve; ΔAUCPR = AUCPR from transfer learning - AUCPR from training from scratch; TL = transfer learning; TF = transcription factor.

**Additional file 7: Fig. S7.** Transfer learning performance using the model architecture of DanQ [46] for the target TFs HNF4A (**A**), JUND (**B**), MAX (**C**), SPI1 (**D**), and SP1 (**E**), from multi-models pre-trained with five TFs with the same binding mode as the target TF (dark blue boxes), five cofactors of the target TF with a different binding mode than the target TF (light blue boxes), five non-cofactors with the same binding mode as the target TF (white boxes), five functional partners of the target TF from STRING with a different binding mode than the target TF (yellow boxes), and five randomly selected TFs with a different binding mode than the target TF (red boxes), with (left) and without (right) the presence of the target TF in the pre-training step. AUCPR = area under the precision-recall curve; BM = binding mode; TF = transcription factor.

**Additional file 8: Fig. S8.** Transfer learning performance using 500-bp long sequences for three target TFs with the same binding mode, but different number of bound regions, from each of the following five families: (**A**) HNF4A, NR2C2, and VD2R (nuclear receptors); (**B**) JUND, ATF7, and NFE2L1 (basic leucine zippers); (**C**) MAX, MNT, and SREBF2 (basic helix-loop-helix factors); (**D**) SPI1, ETV4, and ERG (tryptophan cluster factors); and (**E**) SP1, KLF9, and ZNF740 (C2H2 zinc fingers). For transfer learning, multi-models were pre-trained with five TFs with the same binding mode as the target TF (dark blue boxes), or five randomly selected TFs with a different binding mode than the target TF (red boxes), with (left) and without (right) the presence of the target TF in the pre-training step. The training dataset size of each multi-model is indicated with diamonds (secondary y-axis). The number of bound regions for each TF is shown between parenthesis. The performance of *de novo* PWMs (black stars) is provided as a baseline for each TF. AUCPR = area under the precision-recall curve; BM = binding mode; PWM = position weight matrix; TF = transcription factor.

**Additional file 9: Fig. S9.** Transfer learning performance using 1,000-bp long sequences for three target TFs with the same binding mode, but different number of bound regions, from each of the following five families: (**A**) HNF4A, NR2C2, and VD2R (nuclear receptors); (**B**) JUND, ATF7, and NFE2L1 (basic leucine zippers); (**C**) MAX, MNT, and SREBF2 (basic helix-loop-helix factors); (**D**) SPI1, ETV4, and ERG (tryptophan cluster factors); and (**E**) SP1, KLF9, and ZNF740 (C2H2 zinc fingers). For transfer learning, multi-models were pre-trained with five TFs with the same binding mode as the target TF (dark blue boxes), or five randomly selected TFs with a different binding mode than the target TF (red boxes), with (left) and without (right) the presence of the target TF in the pre-training step. The training dataset size of each multi-model is indicated with diamonds (secondary y-axis). The number of bound regions for each TF is shown between parenthesis. The performance of *de novo* PWMs (black stars) is provided as a baseline for each TF. AUCPR = area under the precision-recall curve; BM = binding mode; PWM = position weight matrix; TF = transcription factor.

**Additional file 10: Fig. S10.** Transfer learning performance for three target TFs with the same binding mode, but different number of bound regions, from each of the following five families: (**A**) HNF4A, NR2C2, and VD2R (nuclear receptors); (**B**) JUND, ATF7, and NFE2L1 (basic leucine zippers); (**C**) MAX, MNT, and SREBF2 (basic helix-loop-helix factors); (**D**) SPI1, ETV4, and ERG (tryptophan cluster factors); and (**E**) SP1, KLF9, and ZNF740 (C2H2 zinc fingers). For transfer learning, multi-models were pre-trained with five cofactors with different binding modes than the target TF (light blue boxes), or five randomly selected TFs with a different binding mode than the target TF (red boxes), with (left) and without (right) the presence of the target TF in the pre-training step. The training dataset size of each multi-model is indicated with diamonds (secondary y-axis). The number of bound regions for each TF is shown between parenthesis. The performance of *de novo* PWMs (black stars) is provided as a baseline for each TF. AUCPR = area under the precision-recall curve; BM = binding mode; PWM = position weight matrix; TF = transcription factor.

**Additional file 11: Fig. S11.** Performance of individual models trained with and without transfer learning for two target TFs with the same binding mode, but different number of bound regions, from each of the following five families: (**A**) NR2C2 and VD2R (nuclear receptors); (**B**) ATF7 and NFE2L1 (basic leucine zippers); (**C**) MNT and SREBF2 (basic helix-loop-helix factors); (**D**) ETV4 and ERG (tryptophan cluster factors); and (**E**) KLF9 and ZNF740 (C2H2 zinc fingers). For transfer learning, multi-models were pre-trained with five TFs with the same binding mode as the target TF, and individual models were fine-tuned using three different initialization strategies: transferring the weights from the first convolutional layer (white boxes); transferring the weights from all convolutional layers (light blue boxes); or transferring the weights from both the convolutional and fully connected layers (except the output layer; dark blue boxes). Individual models without transfer learning were trained from scratch (red boxes) or after initialization using JASPAR profiles and *de novo* PWMs (yellow boxes). The number of bound regions for each TF is shown between parentheses. The performance of *de novo* PWMs (black stars) is provided as a baseline for each TF. AUCPR = area under the precision-recall curve; PWM = position weight matrix; TL = transfer learning; TF = transcription factor.

**Additional file 12: Table S1.** Total number of ones, zeros and nulls in the sparse matrix for the 163 TFs used in this study.

**Additional file 13: Table S2.** Performance for 148 TFs on the original multi-model of 50 TFs, as well as on individual models trained with and without transfer learning. An empty value for multi-model performance indicates that the TF was not used to train the multi-model.

**Additional file 14: Table S3.** List of TF binding modes used in this study.

**Additional file 15: Table S4.** Tomtom similarities of TF profiles from the JASPAR database to the first convolutional layer filters of the original multi-model of 50 TFs, the individual models of HNF4A and SP1, trained with and without transfer learning, and the individual models of JUND trained with transfer learning from multi-models pre-trained on either five cofactors or five random TFs as well as trained from scratch.

**Additional file 16: Table S5.** Transfer learning performance for 148 TFs with and without freezing of the convolutional layers.

**Additional file 17: Table S6.** List of 50 non-redudant JASPAR profiles used to initialize convolutional filter weights.

**Additional file 18.** Review history.

## Data Availability

The TF binding matrices, along with the source code for generating them, are available as 2D numpy arrays [65] on GitHub (https://github.com/wassermanlab/TF-Binding-Matrix [66];). The IPython notebooks and scripts used for the different transfer learning experiments are also available on GitHub (https://github.com/wassermanlab/TF-Binding-Transfer-Learning [67];). The versions used for this manuscript have been deposited in Zenodo [68, 69].

## Abbreviations

AUCPR: Area under the precision-recall curve
BM: Binding mode
ChIP-seq: Chromatin immunoprecipitation followed by sequencing
CNN: Convolutional neural network
DHS: DNase I hypersensitive site
NS: Non-significant
PFM: Position frequency matrix
PWM: Position weight matrix
TL: Transfer learning
TF: Transcription factor
TFBS: Transcription factor binding site

## References

[1] Lovering RC, Gaudet P, Acencio ML, Ignatchenko A, Jolma A, Fornes O, et al. A GO catalogue of human DNA-binding transcription factors. bioRxiv. 2020;2020.10.28.359232 Cold Spring Harbor Laboratory. 10.1016/j.bbagrm.2021.194765 34673265

[2] Mathelier A, Shi W, Wasserman WW (2015). Identification of altered cis-regulatory elements in human disease. Trends Genet.

[3] van der Lee R, Correard S, Wasserman WW (2020). Deregulated regulators: disease-causing cis variants in transcription factor genes. Trends Genet.

[4] Nebert DW (2002). Transcription factors and cancer: an overview. Toxicology..

[5] Khurana E, Fu Y, Chakravarty D, Demichelis F, Rubin MA, Gerstein M (2016). Role of non-coding sequence variants in cancer. Nat Rev Genet..

[6] Johnson DS, Mortazavi A, Myers RM, Wold B (2007). Genome-wide mapping of in vivo protein-DNA interactions. Science.

[7] Chèneby J, Gheorghe M, Artufel M, Mathelier A, Ballester B (2018). ReMap 2018: an updated atlas of regulatory regions from an integrative analysis of DNA-binding ChIP-seq experiments. Nucleic Acids Res..

[8] Chèneby J, Ménétrier Z, Mestdagh M, Rosnet T, Douida A, Rhalloussi W, Bergon A, Lopez F, Ballester B (2020). ReMap 2020: a database of regulatory regions from an integrative analysis of Human and Arabidopsis DNA-binding sequencing experiments. Nucleic Acids Res.x.

[9] Wasserman WW, Sandelin A (2004). Applied bioinformatics for the identification of regulatory elements. Nat Rev Genet..

[10] Gheorghe M, Sandve GK, Khan A, Chèneby J, Ballester B, Mathelier A (2019). A map of direct TF–DNA interactions in the human genome. Nucleic Acids Res..

[11] Snyder MP, Gingeras TR, Moore JE, Weng Z, Gerstein MB, Ren B (2020). Perspectives on ENCODE. Nature..

[12] Koo PK, Ploenzke M. Deep learning for inferring transcription factor binding sites. Curr Opin Syst Biol. 2020; Available from: http://www.sciencedirect.com/science/article/pii/S2452310020300032. [cited 2020 Jul 10]. 10.1016/j.coisb.2020.04.001 PMC7469942 32905524

[13] Weiss K, Khoshgoftaar TM, Wang D (2016). A survey of transfer learning. J Big Data..

[14] Pierson E, Koller D, Battle A, Mostafavi S, Consortium the Gte (2015). Sharing and specificity of co-expression networks across 35 human tissues. PLOS Comput Biol.

[15] Yang Y, Fang Q, Shen H-B (2019). Predicting gene regulatory interactions based on spatial gene expression data and deep learning. PLOS Comput Biol.

[16] Mignone P, Pio G, D’Elia D, Ceci M (2020). Exploiting transfer learning for the reconstruction of the human gene regulatory network. Bioinformatics.

[17] Mieth B, JRF H, Görnitz N, Vidovic MM-C, Müller K-R, Gutteridge A (2019). Using transfer learning from prior reference knowledge to improve the clustering of single-cell RNA-Seq data. Sci Rep.

[18] Wang J, Agarwal D, Huang M, Hu G, Zhou Z, Ye C, Zhang NR (2019). Data denoising with transfer learning in single-cell transcriptomics. Nat Methods.

[19] Wang T, Johnson TS, Shao W, Lu Z, Helm BR, Zhang J, Huang K (2019). BERMUDA: a novel deep transfer learning method for single-cell RNA sequencing batch correction reveals hidden high-resolution cellular subtypes. Genome Biol..

[20] Peng M, Li Y, Wamsley B, Wei Y, Roeder K. Integration and transfer learning of single-cell transcriptomes via cFIT. Proc Natl Acad Sci. 2021;118 [cited 2021 May 28]. National Academy of Sciences;. Available from: https://www.pnas.org/content/118/10/e2024383118. 10.1073/pnas.2024383118 PMC7958425 33658382

[21] Avsec Ž, Kreuzhuber R, Israeli J, Xu N, Cheng J, Shrikumar A, Banerjee A, Kim DS, Beier T, Urban L, Kundaje A, Stegle O, Gagneur J (2019). The Kipoi repository accelerates community exchange and reuse of predictive models for genomics. Nat Biotechnol..

[22] Schwessinger R, Gosden M, Downes D, Brown RC, Oudelaar AM, Telenius J, Teh YW, Lunter G, Hughes JR (2020). DeepC: predicting 3D genome folding using megabase-scale transfer learning. Nat Methods..

[23] Lan G, Zhou J, Xu R, Lu Q, Wang H (2019). Cross-cell-type prediction of TF-binding site by integrating convolutional neural network and adversarial network. Int J Mol Sci.

[24] Zheng A, Lamkin M, Zhao H, Wu C, Su H, Gymrek M (2021). Deep neural networks identify sequence context features predictive of transcription factor binding. Nat Mach Intell..

[25] Deng J, Dong W, Socher R, Li L-J, Li K, Fei-Fei L (2009). ImageNet: a large-scale hierarchical image database. 2009 IEEE Conf Comput Vis Pattern Recognit.

[26] Zeiler MD, Fergus R, Fleet D, Pajdla T, Schiele B, Tuytelaars T (2014). Visualizing and understanding convolutional networks. Comput Vis – ECCV 2014.

[27] Nakato R, Shirahige K (2017). Recent advances in ChIP-seq analysis: from quality management to whole-genome annotation. Brief Bioinform..

[28] Karimzadeh M, Hoffman MM. Virtual ChIP-seq: predicting transcription factor binding by learning from the transcriptome. bioRxiv. 2019:168419 Cold Spring Harbor Laboratory. 10.1186/s13059-022-02690-2 PMC9185870 35681170

[29] Bailey TL, Machanick P (2012). Inferring direct DNA binding from ChIP-seq. Nucleic Acids Res..

[30] Wang J, Zhuang J, Iyer S, Lin X, Whitfield TW, Greven MC, Pierce BG, Dong X, Kundaje A, Cheng Y, Rando OJ, Birney E, Myers RM, Noble WS, Snyder M, Weng Z (2012). Sequence features and chromatin structure around the genomic regions bound by 119 human transcription factors. Genome Res..

[31] Teytelman L, Thurtle DM, Rine J, Oudenaarden AV (2013). Highly expressed loci are vulnerable to misleading ChIP localization of multiple unrelated proteins. Proc Natl Acad Sci.

[32] Worsley Hunt R, Wasserman WW. Non-targeted transcription factors motifs are a systemic component of ChIP-seq datasets. Genome Biol. 2014;15 Available from: https://www.ncbi.nlm.nih.gov/pmc/articles/PMC4165360/. [cited 2020 Jul 21]. 10.1186/s13059-014-0412-4 PMC4165360 25070602

[33] Wreczycka K, Franke V, Uyar B, Wurmus R, Bulut S, Tursun B, Akalin A (2019). HOT or not: examining the basis of high-occupancy target regions. Nucleic Acids Res..

[34] Dror I, Golan T, Levy C, Rohs R, Mandel-Gutfreund Y (2015). A widespread role of the motif environment in transcription factor binding across diverse protein families. Genome Res..

[35] Worsley Hunt R, Mathelier A, del Peso L, Wasserman WW (2014). Improving analysis of transcription factor binding sites within ChIP-Seq data based on topological motif enrichment. BMC Genomics..

[36] Frenkel ZM, Trifonov EN, Volkovich Z, Bettecken T (2011). Nucleosome positioning patterns derived from human apoptotic nucleosomes. J Biomol Struct Dyn.

[37] Zhu F, Farnung L, Kaasinen E, Sahu B, Yin Y, Wei B (2018). The interaction landscape between transcription factors and the nucleosome. Nature..

[38] Eraslan G, Avsec Ž, Gagneur J, Theis FJ (2019). Deep learning: new computational modelling techniques for genomics. Nat Rev Genet..

[39] Wingender E, Schoeps T, Haubrock M, Krull M, Dönitz J (2018). TFClass: expanding the classification of human transcription factors to their mammalian orthologs. Nucleic Acids Res..

[40] Capellera-Garcia S, Pulecio J, Dhulipala K, Siva K, Rayon-Estrada V, Singbrant S, Sommarin MNE, Walkley CR, Soneji S, Karlsson G, Raya Á, Sankaran VG, Flygare J (2016). Defining the minimal factors required for erythropoiesis through direct lineage conversion. Cell Rep..

[41] Lu R, Mucaki EJ, Rogan PK (2017). Discovery and validation of information theory-based transcription factor and cofactor binding site motifs. Nucleic Acids Res..

[42] Szklarczyk D, Gable AL, Lyon D, Junge A, Wyder S, Huerta-Cepas J, Simonovic M, Doncheva NT, Morris JH, Bork P, Jensen LJ, Mering C (2019). STRING v11: protein–protein association networks with increased coverage, supporting functional discovery in genome-wide experimental datasets. Nucleic Acids Res..

[43] Zhou J, Troyanskaya OG (2015). Predicting effects of noncoding variants with deep learning–based sequence model. Nat Methods..

[44] Ambrosini G, Vorontsov I, Penzar D, Groux R, Fornes O, Nikolaeva DD, Ballester B, Grau J, Grosse I, Makeev V, Kulakovskiy I, Bucher P (2020). Insights gained from a comprehensive all-against-all transcription factor binding motif benchmarking study. Genome Biol..

[45] Fornes O, Castro-Mondragon JA, Khan A, van der Lee R, Zhang X, Richmond PA, Modi BP, Correard S, Gheorghe M, Baranašić D, Santana-Garcia W, Tan G, Chèneby J, Ballester B, Parcy F, Sandelin A, Lenhard B, Wasserman WW, Mathelier A (2020). JASPAR 2020: update of the open-access database of transcription factor binding profiles. Nucleic Acids Res..

[46] Quang D, Xie X (2016). DanQ: a hybrid convolutional and recurrent deep neural network for quantifying the function of DNA sequences. Nucleic Acids Res..

[47] Gupta S, Stamatoyannopoulos JA, Bailey TL, Noble WS (2007). Quantifying similarity between motifs. Genome Biol..

[48] Shrikumar A, Greenside P, Kundaje A (2019). Learning important features through propagating activation differences.

[49] Shrikumar A, Tian K, Avsec Ž, Shcherbina A, Banerjee A, Sharmin M (2020). Technical Note on Transcription Factor Motif Discovery from Importance Scores (TF-MoDISco) version 0.5.6.5.

[50] Sandelin A, Wasserman WW (2004). Constrained binding site diversity within families of transcription factors enhances pattern discovery bioinformatics. J Mol Biol..

[51] Smith SL, Kindermans P-J, Ying C, Le QV (2018). Don’t Decay the Learning Rate, Increase the Batch Size.

[52] Kandel I, Castelli M (2020). The effect of batch size on the generalizability of the convolutional neural networks on a histopathology dataset. ICT Express..

[53] Dunham I, Kundaje A, Aldred SF, Collins PJ, Davis CA, Doyle F (2012). An integrated encyclopedia of DNA elements in the human genome. Nature..

[54] Lee CM, Barber GP, Casper J, Clawson H, Diekhans M, Gonzalez JN, Hinrichs AS, Lee BT, Nassar LR, Powell CC, Raney BJ, Rosenbloom KR, Schmelter D, Speir ML, Zweig AS, Haussler D, Haeussler M, Kuhn RM, Kent WJ (2020). UCSC Genome Browser enters 20th year. Nucleic Acids Res..

[55] Quinlan AR, Hall IM (2010). BEDTools: a flexible suite of utilities for comparing genomic features. Bioinformatics..

[56] Pedregosa F, Varoquaux G, Gramfort A, Michel V, Thirion B, Grisel O (2011). Scikit-learn: machine learning in python. J Mach Learn Res..

[57] Kelley DR, Snoek J, Rinn JL (2016). Basset: learning the regulatory code of the accessible genome with deep convolutional neural networks. Genome Res..

[58] Maslova A, Ramirez RN, Ma K, Schmutz H, Wang C, Fox C, Ng B, Benoist C, Mostafavi S, Immunological Genome Project (2020). Deep learning of immune cell differentiation. Proc Natl Acad Sci..

[59] Paszke A, Gross S, Massa F, Lerer A, Bradbury J, Chanan G (2019). PyTorch: an imperative style, high-performance deep learning library. Adv Neural Inf Process Syst..

[60] Kingma DP, Ba J (2017). Adam: a method for stochastic optimization.

[61] Kokhlikyan N, Miglani V, Martin M, Wang E, Alsallakh B, Reynolds J (2020). Captum: a unified and generic model interpretability library for PyTorch.

[62] Bailey TL. STREME: accurate and versatile sequence motif discovery. Bioinformatics. 2021 [cited 2021 May 13]; Available from:. 10.1093/bioinformatics/btab203. 10.1093/bioinformatics/btab203 PMC8479671 33760053

[63] Ambrosini G, Groux R, Bucher P (2018). PWMScan: a fast tool for scanning entire genomes with a position-specific weight matrix. Bioinformatics..

[64] Cock PJA, Antao T, Chang JT, Chapman BA, Cox CJ, Dalke A, Friedberg I, Hamelryck T, Kauff F, Wilczynski B, de Hoon MJL (2009). Biopython: freely available Python tools for computational molecular biology and bioinformatics. Bioinformatics..

[65] Harris CR, Millman KJ, van der Walt SJ, Gommers R, Virtanen P, Cournapeau D (2020). Array programming with NumPy. Nature..

[66] Novakovsky G, Saraswat M, Fornes O, Mostafavi S, Wasserman WW. Biologically-relevant transfer learning improves transcription factor binding prediction: TF binding matrices: GitHub; 2021. Available from: https://github.com/wassermanlab/TF-Binding-Matrix 10.1186/s13059-021-02499-5 PMC8474956 34579793

[67] Novakovsky G, Saraswat M, Fornes O, Mostafavi S, Wasserman WW. Biologically-relevant transfer learning improves transcription factor binding prediction: IPython notebooks and scripts: GitHub; 2021. Available from: https://github.com/wassermanlab/TF-Binding-Transfer-Learning 10.1186/s13059-021-02499-5 PMC8474956 34579793

[68] Novakovsky G, Saraswat M, Fornes O, Mostafavi S, Wasserman WW. Biologically-relevant transfer learning improves transcription factor binding prediction: TF binding matrices: Zenodo; 2021. Available from:. 10.5281/zenodo.5283416. 10.1186/s13059-021-02499-5 PMC8474956 34579793

[69] Novakovsky G, Saraswat M, Fornes O, Mostafavi S, Wasserman WW. Biologically-relevant transfer learning improves transcription factor binding prediction: IPython notebooks and scripts: Zenodo; 2021. Available from:. 10.5281/zenodo.5295097. 10.1186/s13059-021-02499-5 PMC8474956 34579793

