# Supplementary figures and images for: Biologically relevant transfer learning improves transcription factor binding prediction
Source: Genome Biol. 2021 Sep 27;22:280. doi: 10.1186/s13059-021-02499-5 (PMC8474956; doi:10.1186/s13059-021-02499-5)

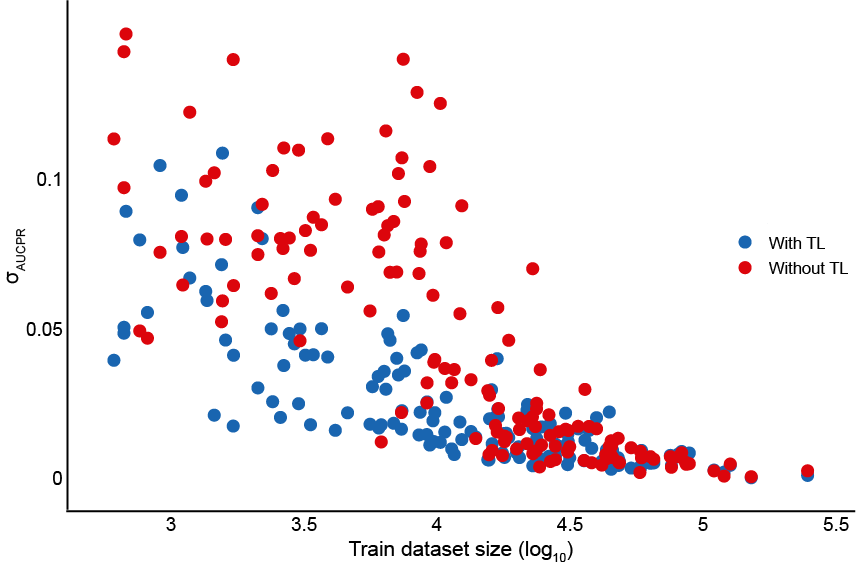

Supplement: Supplementary file 1 — Additional file 1: Fig. S1. Performance variance (i.e., σ; y-axis) by means of AUCPR of individual models trained with (blue) and without transfer learning (red) is plotted with respect to the size of the training dataset (x-axis) for 148 TFs. AUCPR = area under the precision-recall curve; TF = transcription factor; TL = transfer learning. [file 13059_2021_2499_MOESM1_ESM.png]

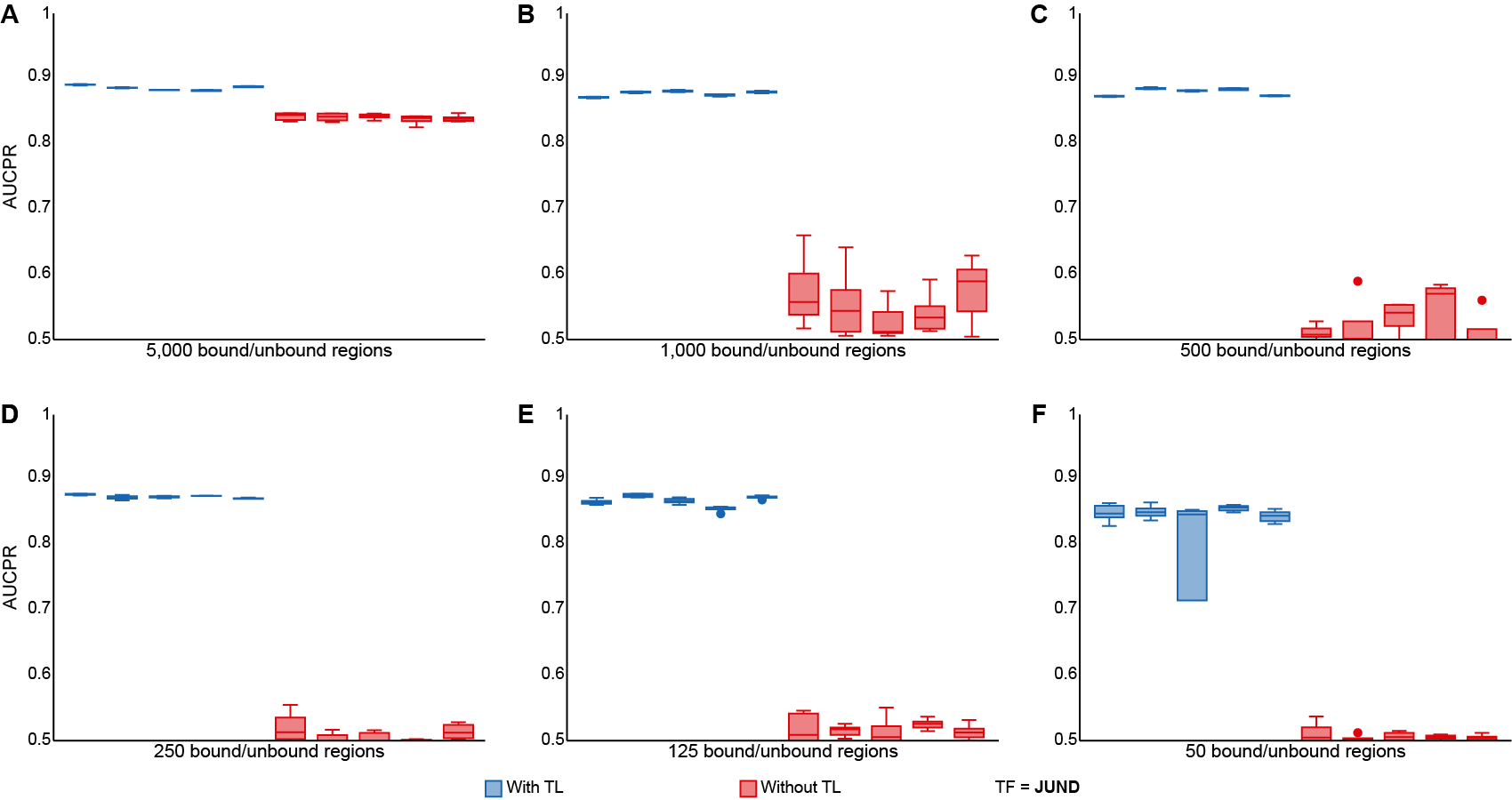

Supplement: Supplementary file 2 — Additional file 2: Fig. S2. Performance of JUND models trained with (blue boxes) and without (red boxes) transfer learning on 5,000 (A), 1,000 (B), 500 (C), 250 (D), 125 (E), and 50 (F), bound and unbound regions. Each model was trained five times with different random initializations to ensure the robustness of the results. AUCPR = area under the precision-recall curve; TF = transcription factor; TL = transfer learning. [file 13059_2021_2499_MOESM2_ESM.png]

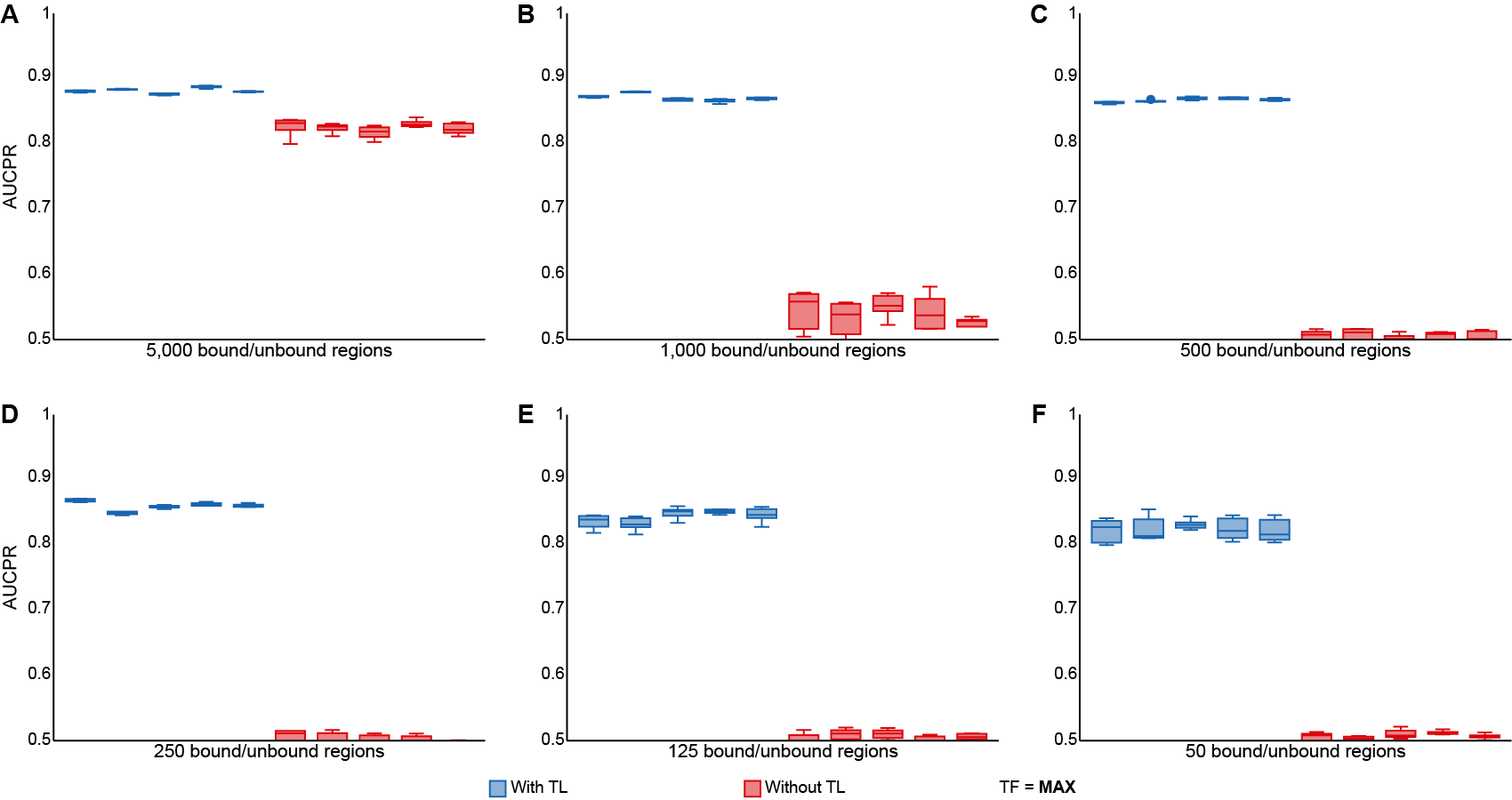

Supplement: Supplementary file 3 — Additional file 3: Fig. S3. Performance of MAX models trained with (blue boxes) and without (red boxes) transfer learning on 5,000 (A), 1,000 (B), 500 (C), 250 (D), 125 (E), and 50 (F), bound and unbound regions. Each model was trained five times with different random initializations to ensure the robustness of the results. AUCPR = area under the precision-recall curve; TF = transcription factor; TL = transfer learning. [file 13059_2021_2499_MOESM3_ESM.png]

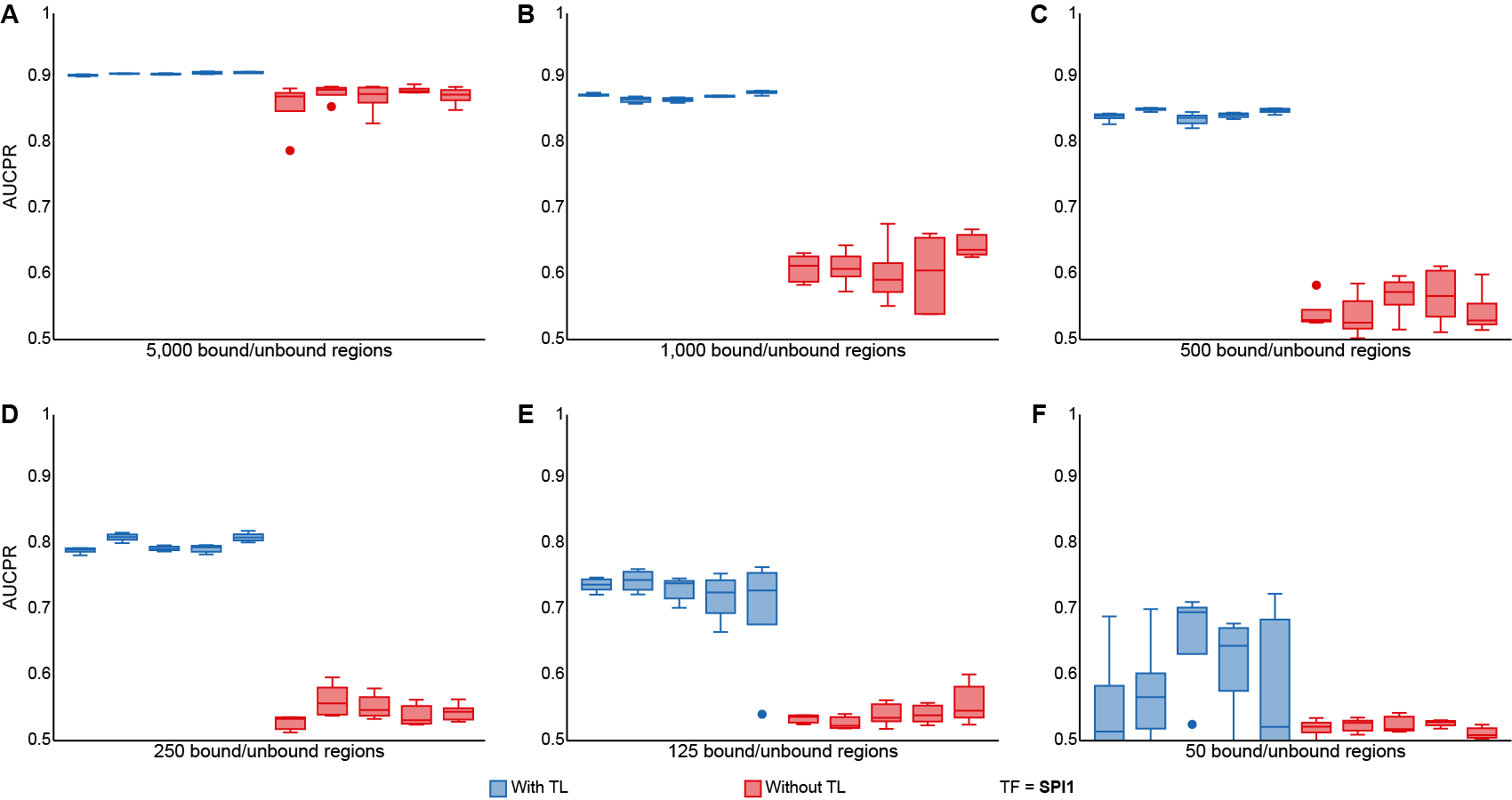

Supplement: Supplementary file 4 — Additional file 4: Fig. S4. Performance of SPI1 models trained with (blue boxes) and without (red boxes) transfer learning on 5,000 (A), 1,000 (B), 500 (C), 250 (D), 125 (E), and 50 (F), bound and unbound regions. Each model was trained five times with different random initializations to ensure the robustness of the results. AUCPR = area under the precision-recall curve; TF = transcription factor; TL = transfer learning. [file 13059_2021_2499_MOESM4_ESM.png]

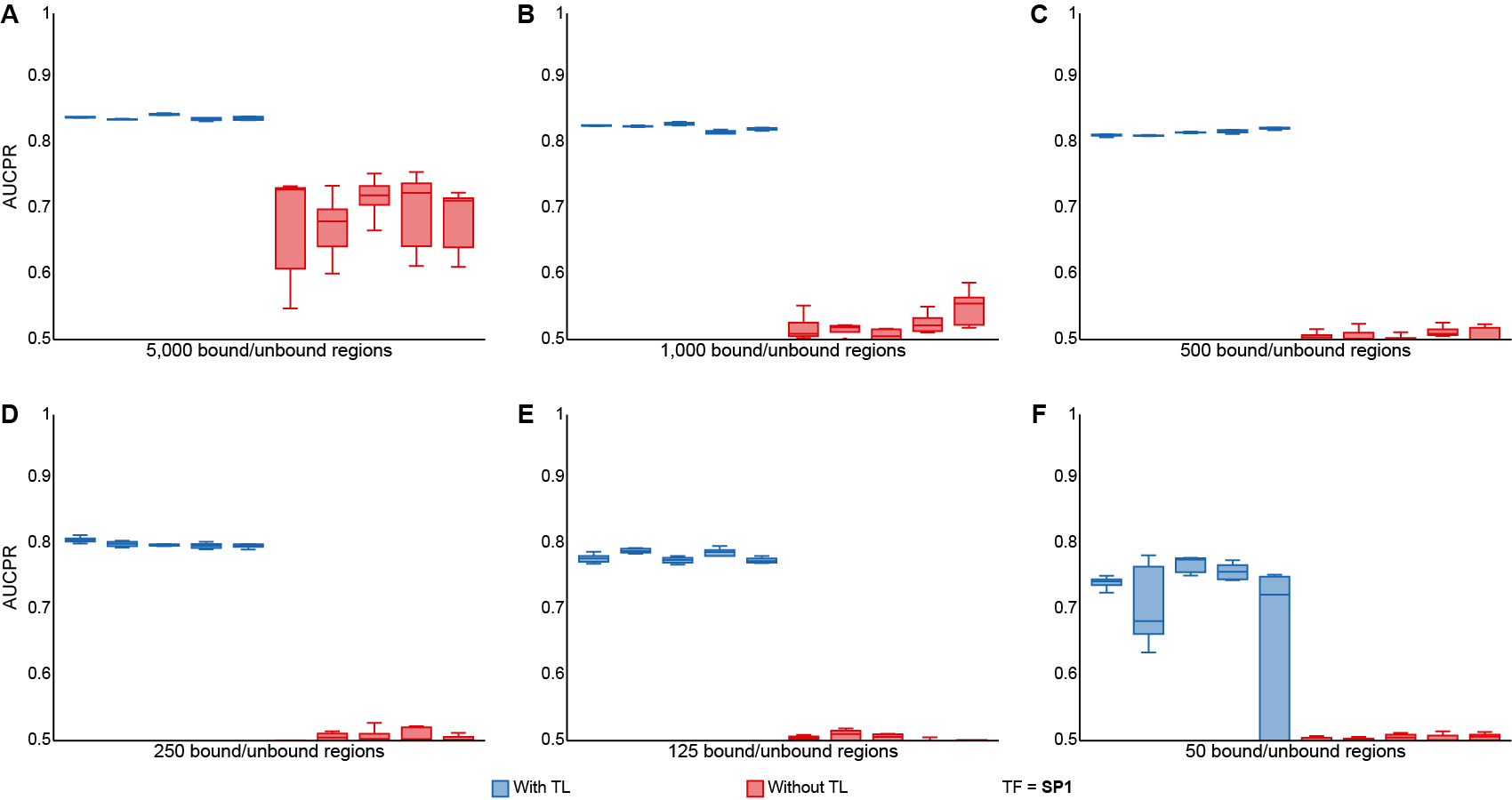

Supplement: Supplementary file 5 — Additional file 5: Fig. S5. Performance of SP1 models trained with (blue boxes) and without (red boxes) transfer learning on 5,000 (A), 1,000 (B), 500 (C), 250 (D), 125 (E), and 50 (F), bound and unbound regions. Each model was trained five times with different random initializations to ensure the robustness of the results. AUCPR = area under the precision-recall curve; TF = transcription factor; TL = transfer learning. [file 13059_2021_2499_MOESM5_ESM.png]

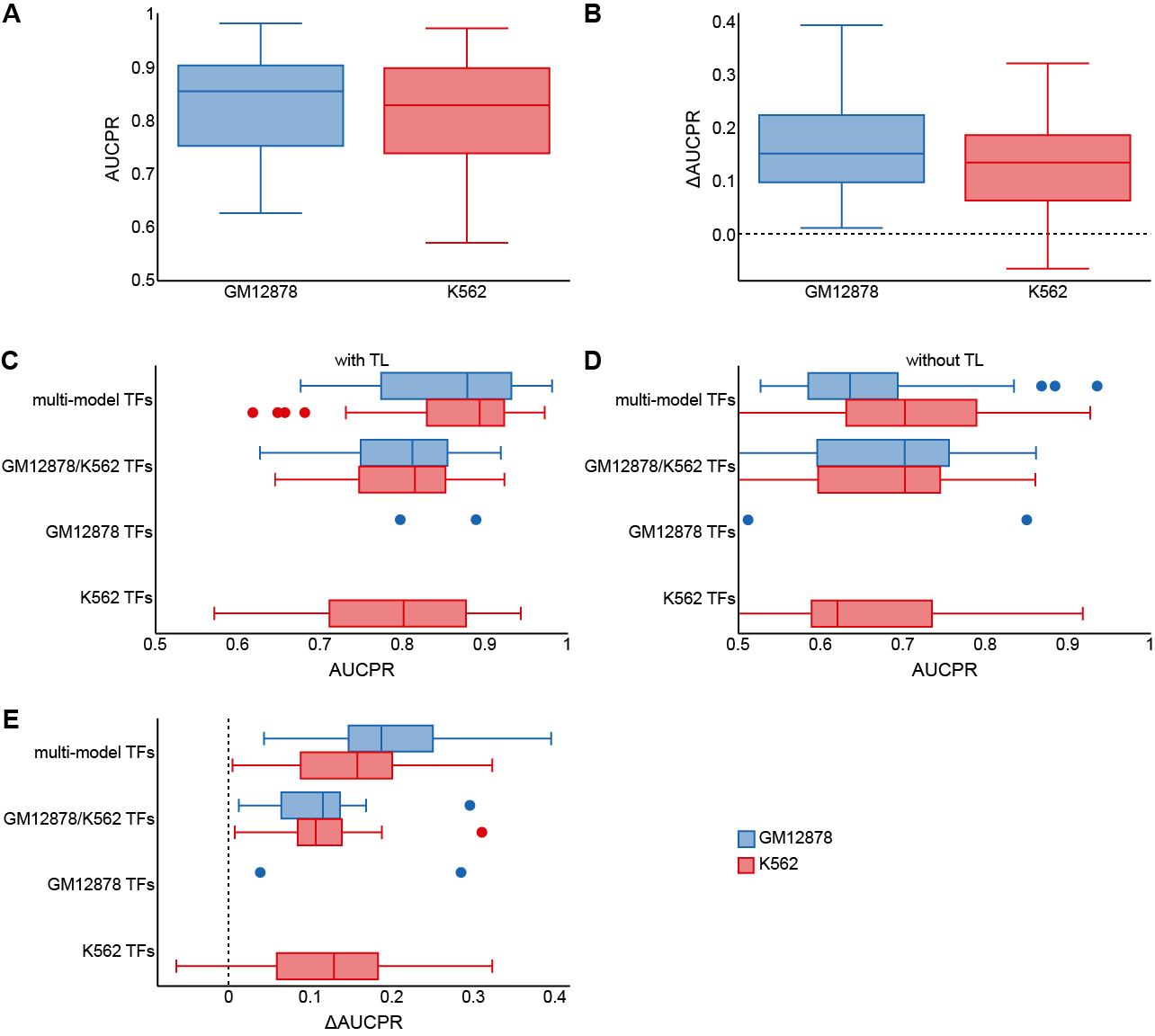

Supplement: Supplementary file 6 — Additional file 6: Fig. S6. (A) Performance of transfer learning models trained on either GM12878 (blue boxes) or K562 cell data (red boxes). (B) Performance difference (i.e., ΔAUCPR) of individual models trained with and without transfer learning on either GM12878 or K562 cell data. Performance of individual models trained with (C) and without (D) transfer learning on either GM12878 or K562 cell data for 35 multi-model TFs, as well as for 76 additional TFs with resolved regions in either GM12878 cells (2), K562 cells (55), or both (19; i.e., GM12878/K562 TFs). (E) Performance difference of individual models trained with and without transfer learning on GM12878 or K562 cell data for the previous TF categories. Transfer learning models were pre-trained using data from GM12878 cells. AUCPR = area under the precision-recall curve; ΔAUCPR = AUCPR from transfer learning - AUCPR from training from scratch; TL = transfer learning; TF = transcription factor. [file 13059_2021_2499_MOESM6_ESM.png]

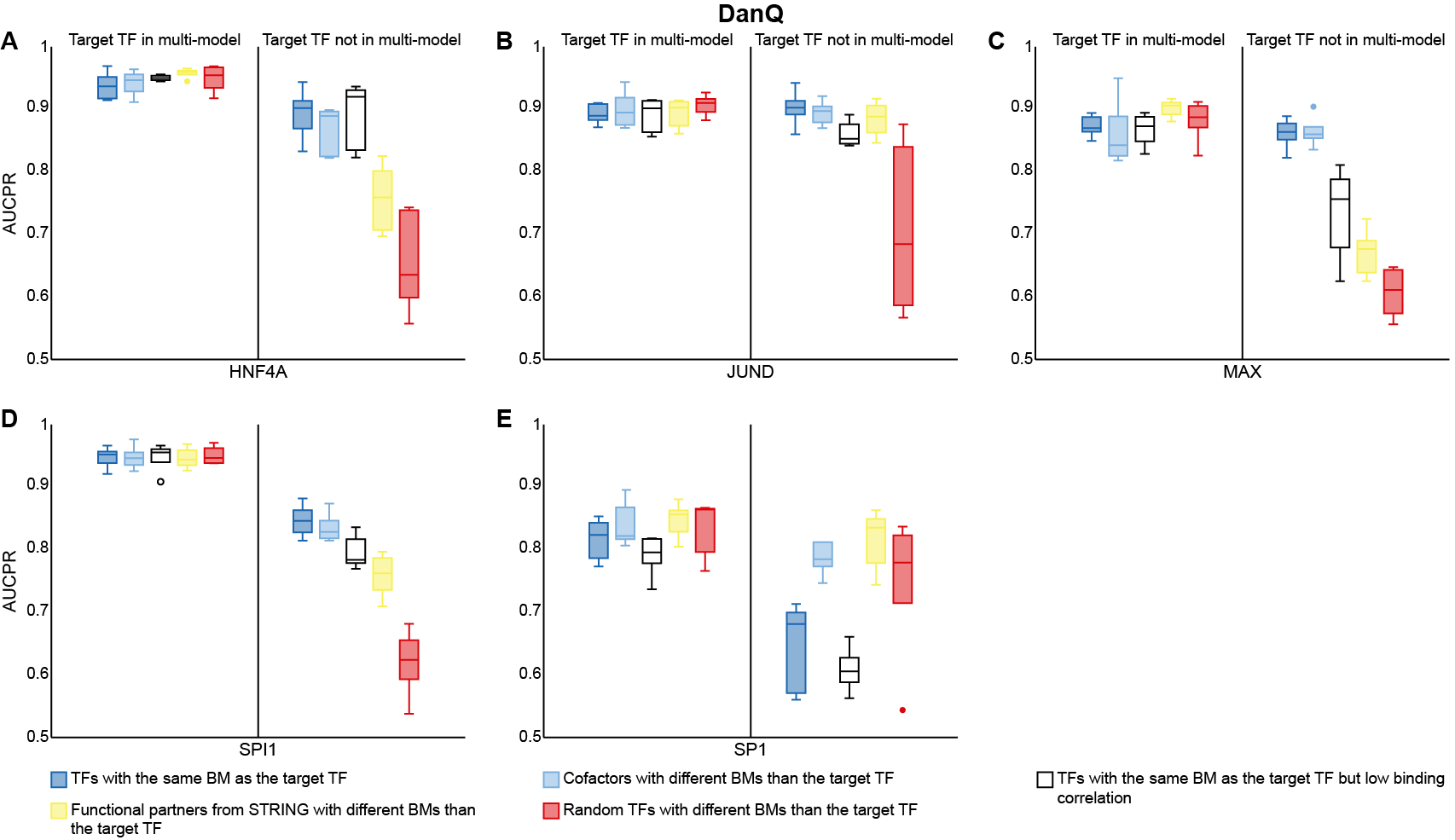

Supplement: Supplementary file 7 — Additional file 7: Fig. S7. Transfer learning performance using the model architecture of DanQ [46] for the target TFs HNF4A (A), JUND (B), MAX (C), SPI1 (D), and SP1 (E), from multi-models pre-trained with five TFs with the same binding mode as the target TF (dark blue boxes), five cofactors of the target TF with a different binding mode than the target TF (light blue boxes), five non-cofactors with the same binding mode as the target TF (white boxes), five functional partners of the target TF from STRING with a different binding mode than the target TF (yellow boxes), and five randomly selected TFs with a different binding mode than the target TF (red boxes), with (left) and without (right) the presence of the target TF in the pre-training step. AUCPR = area under the precision-recall curve; BM = binding mode; TF = transcription factor. [file 13059_2021_2499_MOESM7_ESM.png]

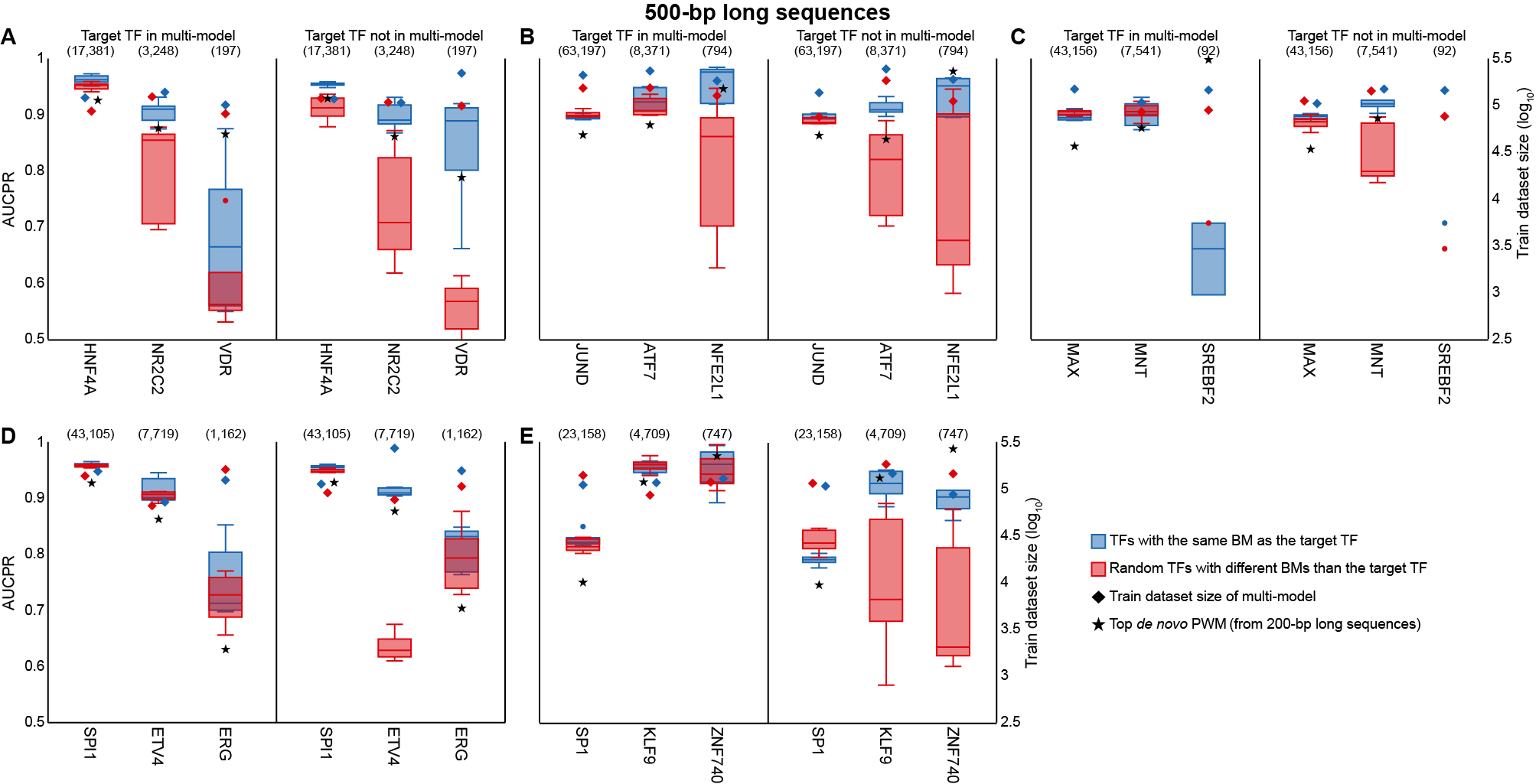

Supplement: Supplementary file 8 — Additional file 8: Fig. S8. Transfer learning performance using 500-bp long sequences for three target TFs with the same binding mode, but different number of bound regions, from each of the following five families: (A) HNF4A, NR2C2, and VD2R (nuclear receptors); (B) JUND, ATF7, and NFE2L1 (basic leucine zippers); (C) MAX, MNT, and SREBF2 (basic helix-loop-helix factors); (D) SPI1, ETV4, and ERG (tryptophan cluster factors); and (E) SP1, KLF9, and ZNF740 (C2H2 zinc fingers). For transfer learning, multi-models were pre-trained with five TFs with the same binding mode as the target TF (dark blue boxes), or five randomly selected TFs with a different binding mode than the target TF (red boxes), with (left) and without (right) the presence of the target TF in the pre-training step. The training dataset size of each multi-model is indicated with diamonds (secondary y-axis). The number of bound regions for each TF is shown between parenthesis. The performance of de novo PWMs (black stars) is provided as a baseline for each TF. AUCPR = area under the precision-recall curve; BM = binding mode; PWM = position weight matrix; TF = transcription factor. [file 13059_2021_2499_MOESM8_ESM.png]

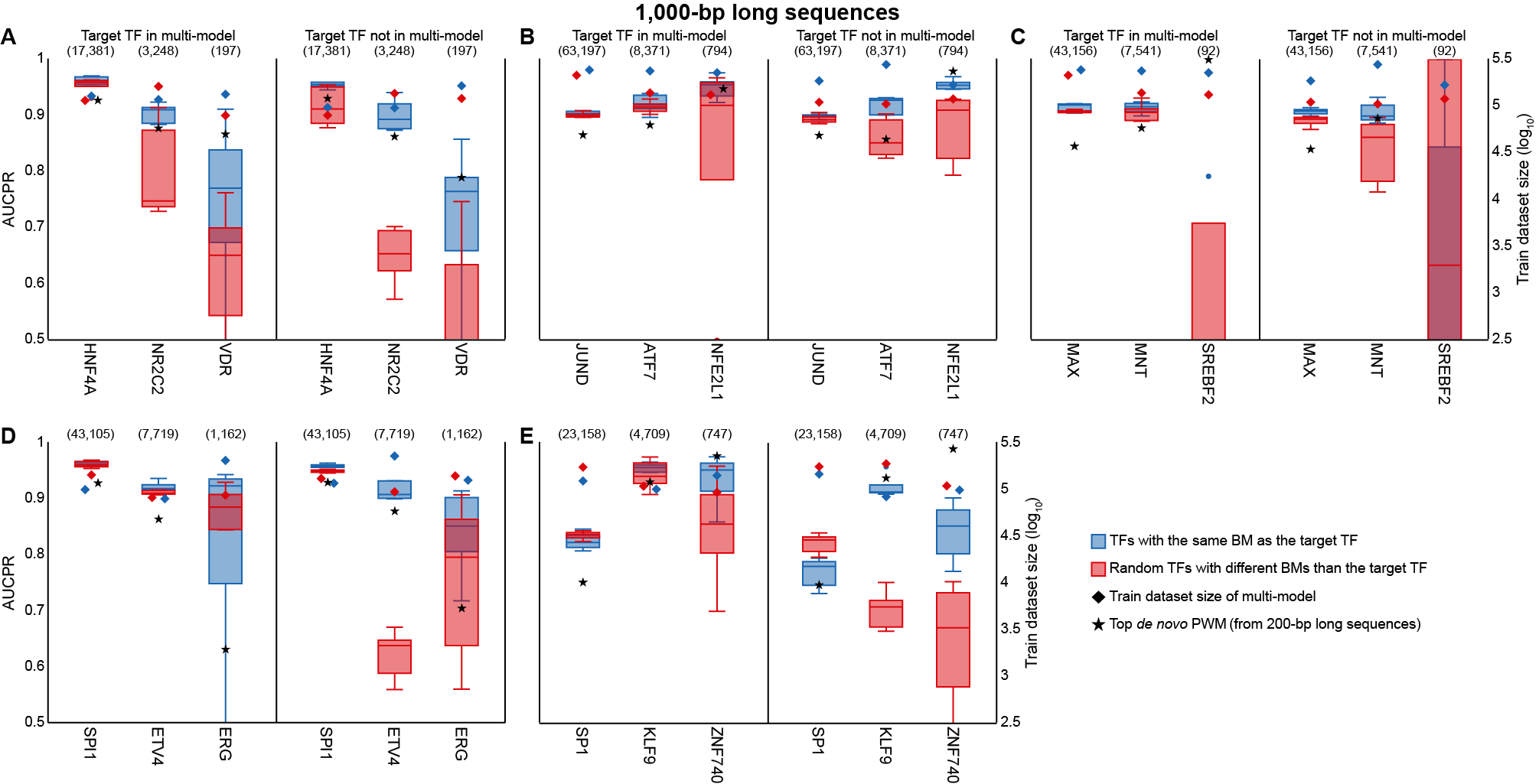

Supplement: Supplementary file 9 — Additional file 9: Fig. S9. Transfer learning performance using 1,000-bp long sequences for three target TFs with the same binding mode, but different number of bound regions, from each of the following five families: (A) HNF4A, NR2C2, and VD2R (nuclear receptors); (B) JUND, ATF7, and NFE2L1 (basic leucine zippers); (C) MAX, MNT, and SREBF2 (basic helix-loop-helix factors); (D) SPI1, ETV4, and ERG (tryptophan cluster factors); and (E) SP1, KLF9, and ZNF740 (C2H2 zinc fingers). For transfer learning, multi-models were pre-trained with five TFs with the same binding mode as the target TF (dark blue boxes), or five randomly selected TFs with a different binding mode than the target TF (red boxes), with (left) and without (right) the presence of the target TF in the pre-training step. The training dataset size of each multi-model is indicated with diamonds (secondary y-axis). The number of bound regions for each TF is shown between parenthesis. The performance of de novo PWMs (black stars) is provided as a baseline for each TF. AUCPR = area under the precision-recall curve; BM = binding mode; PWM = position weight matrix; TF = transcription factor. [file 13059_2021_2499_MOESM9_ESM.png]

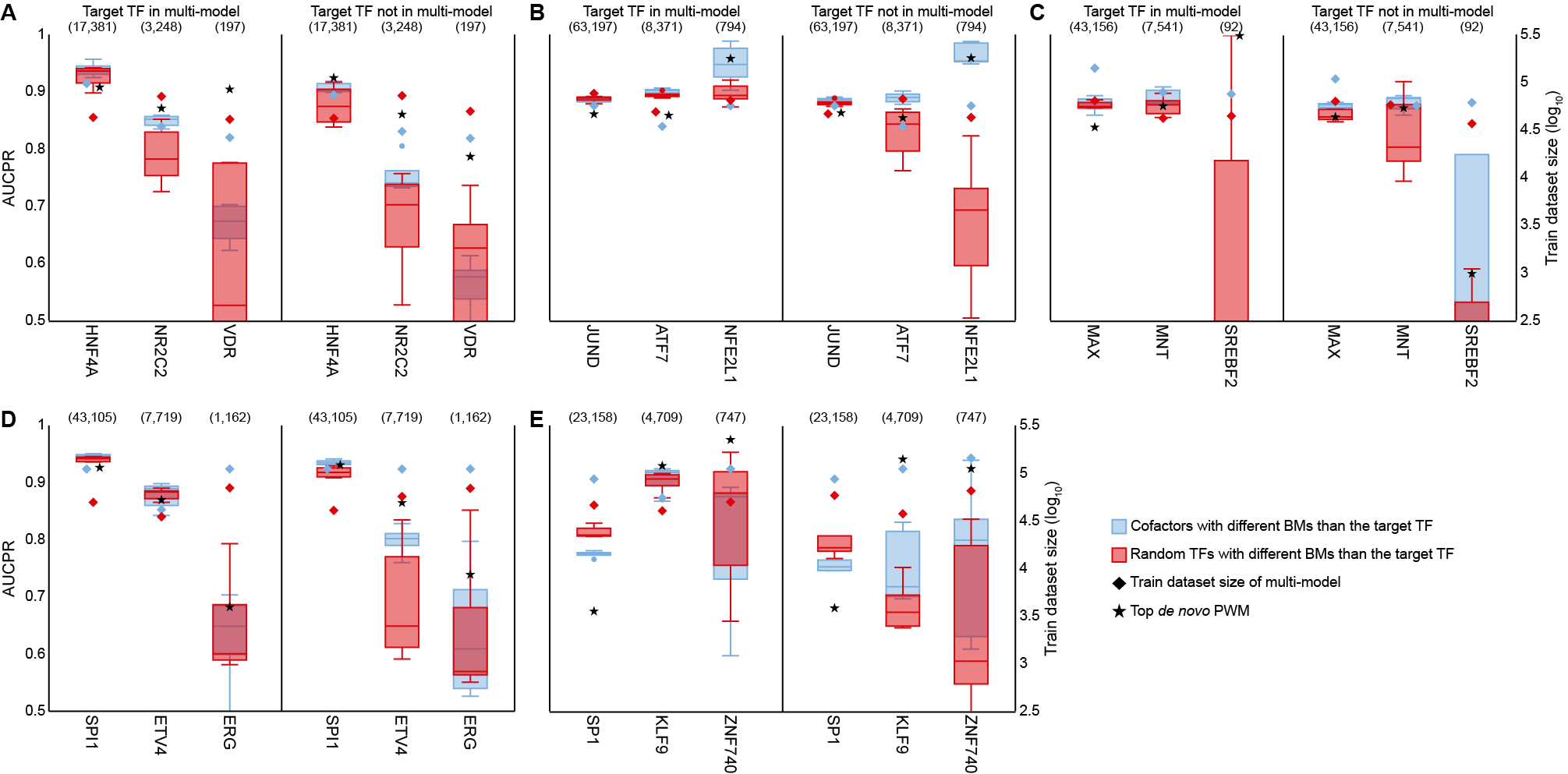

Supplement: Supplementary file 10 — Additional file 10: Fig. S10. Transfer learning performance for three target TFs with the same binding mode, but different number of bound regions, from each of the following five families: (A) HNF4A, NR2C2, and VD2R (nuclear receptors); (B) JUND, ATF7, and NFE2L1 (basic leucine zippers); (C) MAX, MNT, and SREBF2 (basic helix-loop-helix factors); (D) SPI1, ETV4, and ERG (tryptophan cluster factors); and (E) SP1, KLF9, and ZNF740 (C2H2 zinc fingers). For transfer learning, multi-models were pre-trained with five cofactors with different binding modes than the target TF (light blue boxes), or five randomly selected TFs with a different binding mode than the target TF (red boxes), with (left) and without (right) the presence of the target TF in the pre-training step. The training dataset size of each multi-model is indicated with diamonds (secondary y-axis). The number of bound regions for each TF is shown between parenthesis. The performance of de novo PWMs (black stars) is provided as a baseline for each TF. AUCPR = area under the precision-recall curve; BM = binding mode; PWM = position weight matrix; TF = transcription factor. [file 13059_2021_2499_MOESM10_ESM.png]

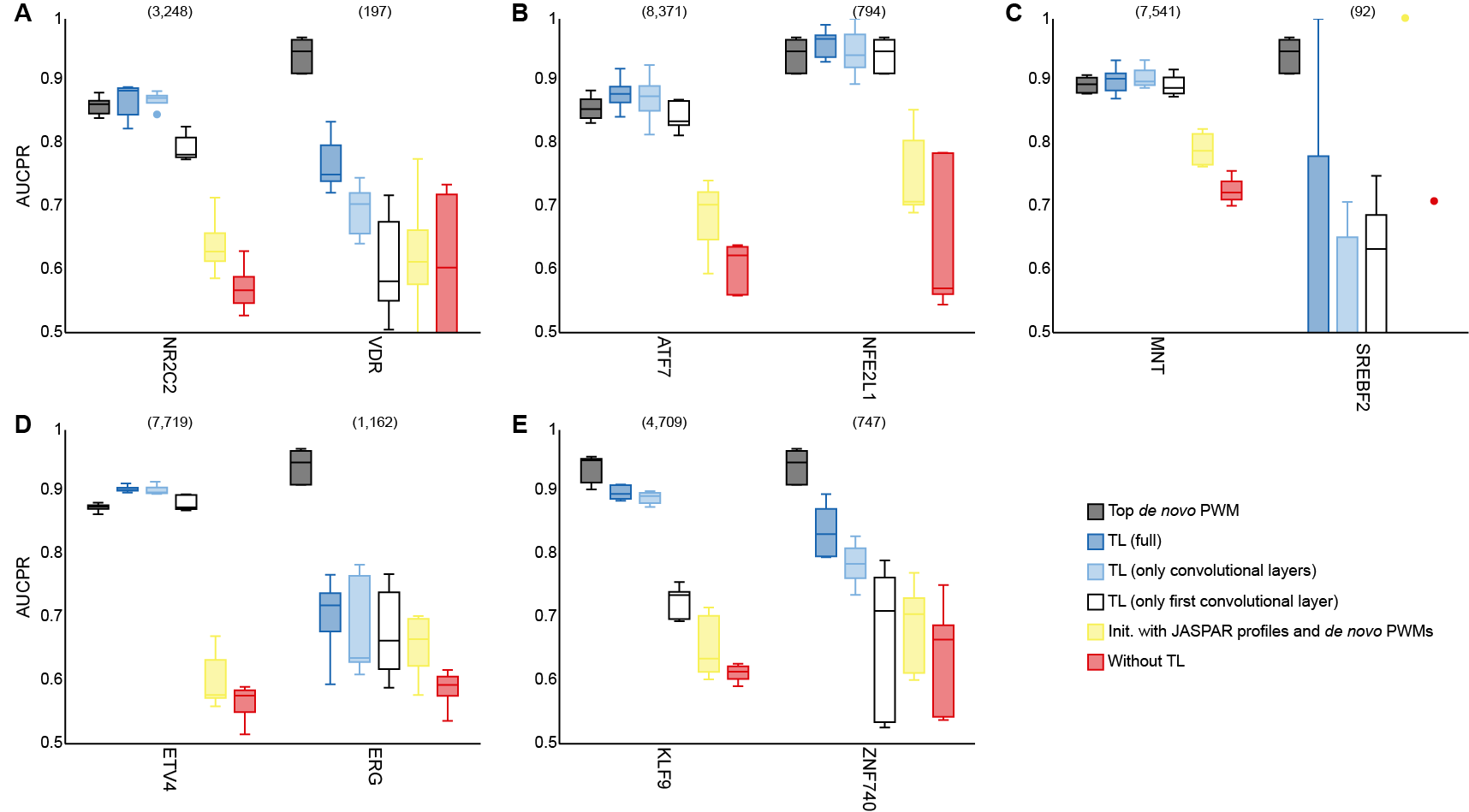

Supplement: Supplementary file 11 — Additional file 11: Fig. S11. Performance of individual models trained with and without transfer learning for two target TFs with the same binding mode, but different number of bound regions, from each of the following five families: (A) NR2C2 and VD2R (nuclear receptors); (B) ATF7 and NFE2L1 (basic leucine zippers); (C) MNT and SREBF2 (basic helix-loop-helix factors); (D) ETV4 and ERG (tryptophan cluster factors); and (E) KLF9 and ZNF740 (C2H2 zinc fingers). For transfer learning, multi-models were pre-trained with five TFs with the same binding mode as the target TF, and individual models were fine-tuned using three different initialization strategies: transferring the weights from the first convolutional layer (white boxes); transferring the weights from all convolutional layers (light blue boxes); or transferring the weights from both the convolutional and fully connected layers (except the output layer; dark blue boxes). Individual models without transfer learning were trained from scratch (red boxes) or after initialization using JASPAR profiles and de novo PWMs (yellow boxes). The number of bound regions for each TF is shown between parentheses. The performance of de novo PWMs (black stars) is provided as a baseline for each TF. AUCPR = area under the precision-recall curve; PWM = position weight matrix; TL = transfer learning; TF = transcription factor. [file 13059_2021_2499_MOESM11_ESM.png]
