# Supplementary material for: Biologically relevant transfer learning improves transcription factor binding prediction
Source: Genome Biol. 2021 Sep 27;22:280. doi: 10.1186/s13059-021-02499-5 (PMC8474956; doi:10.1186/s13059-021-02499-5)
Supplement: Supplementary file 18 — Additional file 18. Review history. [file 13059_2021_2499_MOESM18_ESM.docx]

**Review history**

**First round of review**

**Reviewer 1**

The manuscript "Biologically-relevant transfer learning improves transcription factor binding prediction" by Novakovsky et al. tries to assess a transfer learning strategy to improve the prediction of transcription factor (TF) binding by using deep learning. The authors first train a multi-class classification model on 200 bp regions obtained by combining DHS data from encode, ChIP-seq peaks from ReMap and TFBS from UniBind for multiple TFs. Then, they fine-tune the model on individual TFs separately by transferring the weights of the initial multi-task model to single-task models. The authors investigate how different combinations of TFs in the initial multi-task model affect the performance of the fine-tuned model. This is a timely and interesting technical study, and provides insight into the different components and steps when using deep learning to predict true TFBS. Transfer learning itself has been applied before to this problem (and cited by the authors), thus the main novelty seems to lie in the strategy of how to use the multi-model (e.g., which TFs to select). Although there is no clear biological follow-up in this work, such as new biological insight or experimental validation, the work is useful for the field of gene regulation bioinformatics.

Comments:
1.     When the unified data matrix is built, using the combination of ReMAP, DHS and UniBind seems like a good strategy, however, this leads to using different cell types/tissues for different TFs. Can authors elaborate on this problem and also do control experiments regarding this? (i.e. showing transfer learning performance by choosing a cell type and changing the TFs and/or chosing a TF and changing the cell type)
2.     In many of the TFBS prediction models in the literature, size of the input regions are around 500bp to 1000bp. Did the authors try different sizes? If so, showing the performance of transfer learning regarding the input size would be informative. If not, elaborating on the selection of 200bp is needed in the manuscript.
3.     All individual models with transfer learning are actually trained further with a lower learning rate. For a fair comparison, the multi-task models also should be further trained with a lower learning rate. In addition, the single-task models without transfer learning should be first trained with a higher learning rate (as in multi-model) and then with a lower learning rate.
4.     The performance of the multi-task models are just shown in Figure 3-B. For the sake of fair and better comparison, the performance of the initial multi-task models can be added to the other figures too.
5.     It is known that deep learning models need huge amounts of data to have decent learning. Similar to what the authors show, when there is less data, the performance of the models gets worse. In order to have a good negative control to measure baseline performance, the authors can use only PWMs to differentiate bound and unbound regions for individual TFs and calculate MCC. Particularly, a new PWM can be constructed ('trained') from the positive training data, while leaving out a subset of the positives. Using PWM-scanning with different thresholds on the left-out positives, versus negatives, the AUROC/AUPR can be calculated, or similarly to the deep learning models, the MCC can be compared (for example at the optimal threshold from the AUPR). This baseline is essential, to understand the performance (MCC) of both the multi-model and the fine-tuned models.
6.     The authors show that after the fine-tuning step, certain filters from the first convolutional layer represent binding motifs of the TF used for the fine-tuning. Can these filters be obtained by only using transfer learning? The authors can check the individual models without transfer learning whether the models can catch the binding sites of the related TF in their first convolutional layer.
7.     Measuring the performance of multi-task models can be elaborated, it is not clear from the text.
8.     For the multi-task models, when the sample size of different classes are imbalanced, deep learning models tend to focus on the classes with the higher number of samples. This leads to poor performance on the classes with lower numbers of samples. In order to overcome this problem, using "class_weight" is suggested when training deep learning models. Did the authors try using the class_weight option when training the models?
9.     The authors mention that using DanQ architecture gives better performance, but throughout the whole paper another architecture is used. Can the authors elaborate on the selecting of the architecture used in this paper?
10.     Overall, if I understood correctly, when there is a TF with a similar binding mode to the final TF (or a cofactor of final TF) in the pre-trained multi-model, the transfer learning approach is more useful and increases the performance of the model significantly. This can be explained by the fact that convolutional filters have learned the binding motif of the final TF during the pre-training step. Indeed, the authors observe that freezing the convolution layers leads to decrease in the performance when applying transfer learning strategy. This in fact suggests that the most important part of the model, and of the multi-to-finetuning strategy, are the convolutional layers. These results suggest that inserting PWMs to the convolutional layers prior to training the 1-TF model may have an huge impact on the performance of the model and may even outperform the incremental effect of transfer learning. Therefore, we suggest testing a 1 TF model using Jaspar PWMs as convolutional filters. If such a strategy works as efficiently, then can we conclude that the major contribution of the multi-model step (and the transfer learning), is to set the priors correctly? This seems to be a critical thing to assess, given that this aspect forms the core of the paper/novelty.

**Reviewer 2**

The authors investigate the problem of learning TF binding predictions from Chip-seq data for many different TFs using Deep neural networks with transfer learning. This paper is very timely as the use of transfer learning is becoming very prominent and the observations are of interest. The paper is well written and the experiments are sound.
The authors cite a preprint (ref 24, which is got publised in Nature Machine Intelligence early 2021), which has similairity in that a similar transfer learning strategy is used. However, I personally find that there is not a lot of overlap between the two papers, and that the manuscript here is much more interesting for biologists and computational biologists. In particular the analysis concerning the similarity of binding sites and protein domains, is something which likely extrapolates to related problems, such as the prediction of RNA-binding proteins.

However, there are a number of points that need to be addressed to improve the reading of the manuscript:

Major revision:

1. The authors propose a transfer learning approach to predict TFBS from DNA sequence. Their design is very similar to the newly published work AgentBind, "Zheng et.al., nature machine intelligence, 2021", which they also mention in their paper by stating that they corroborate the findings of Zheng and colleagues. I would find it helpful if the authors would state differences to their study and the other work more clearly (number of TFs, analysis goals etc.).

2. Regarding creating the data, they define three different states based on the binding status of TFs in DNase I hypersensitive sites (DHS). They define state "null" when the binding of the TF to the DHS could not be resolved, which is different than state "0" where DHS was accessible but not bound by the TF. The number of null cases they identified in their data is more than 80%. Since this number was really high, they decided to use a cosine-base similarity metric  combined with MCC (Mathew's correlation coefficient) for their assessment of transfer learning. They show the AUROC and AUCPR on the MCC values. But, I'm not sure why they didn't show the AUROC and AUCPR results directly on the output of their model. Was it because they formulated their problem into three states, instead of two(bound and unbound), or they wanted to avoid showing these metric due to the imbalance nature of the data? Perhaps they could elaborate on these points and otherwise show also AUCPR to show that their general conclusions hold as well using this established metric.

3. It is not clear for the different experiments they did, what the exact input TFs were. For example.  in page 6, lines 18-21, where do they get the additional 99 TFs from? How come they were not used in training the multi-model? It would be helpful if the authors define different input sets that are used for the experiments and then refer to them in a systematic way. in particular it needs to be made very clear, if the same TF was used in the multi-task step or not.

4. The usage of the term "transfer learning" is sometimes a but unclear in the context. In page 8, line 60 they state "... inadequate set of
weights with which to initialize the individual model...". Is it just initializing or really transfer and freezing the weights?
In other words, to this point it's not clear if they modify the weights after initializing using the multi-model step. But, when reading the "transfer learning" section in "Methods", they state that in the fine-tuning step they either train the entire model or just the fully connected layers. Which again it is not clear which case they use. It needs to be made very clear what is freezed when using transfer learning and what not. It should be straightforward to introduce a different learning setups to differentiate between those modes and then properly refer to them in the text.

**Authors’ response to reviewers**

**Reviewer #1**: The manuscript "Biologically-relevant transfer learning improves transcription factor binding prediction" by Novakovsky et al. tries to assess a transfer learning strategy to improve the prediction of transcription factor (TF) binding by using deep learning. The authors first train a multi-class classification model on 200 bp regions obtained by combining DHS data from encode, ChIP-seq peaks from ReMap and TFBS from UniBind for multiple TFs. Then, they fine-tune the model on individual TFs separately by transferring the weights of the initial multi-task model to single-task models. The authors investigate how different combinations of TFs in the initial multi-task model affect the performance of the fine-tuned model. This is a timely and interesting technical study, and provides insight into the different components and steps when using deep learning to predict true TFBS. Transfer learning itself has been applied before to this problem (and cited by the authors), thus the main novelty seems to lie in the strategy of how to use the multi-model (e.g., which TFs to select). Although there is no clear biological follow-up in this work, such as new biological insight or experimental validation, the work is useful for the field of gene regulation bioinformatics.

We appreciate the reviewer’s interest in the manuscript and thank them for the insightful comments.

1. When the unified data matrix is built, using the combination of ReMAP, DHS and UniBind seems like a good strategy, however, this leads to using different cell types/tissues for different TFs. Can authors elaborate on this problem and also do control experiments regarding this? (i.e. showing transfer learning performance by choosing a cell type and changing the TFs and/or chosing a TF and changing the cell type).

This is a good point that we did not consider when we designed the TF binding matrix. As stated in the manuscript (see the first paragraph of the results section “A sparse matrix of TF binding events across accessible genomic regions”), different reports have adopted different definitions for positive and negative regions with which to train deep learning models, each with intrinsic limitations. We therefore designed our TF binding matrix so that it would store reliable positive and negative regions. We used the DNase I hypersensitive site (DHS) data as an atlas of accessible regions onto which we mapped TF binding data from ReMap and UniBind. This resulted in a 3D matrix of 1,817,918 bound and open regions across 163 TFs and 52 cell and tissue types. By aggregating the data in a cell and tissue type-agnostic manner (Fig. 1), we obtained the “sparse” and “less-sparse” matrices that we have used in this work. The main reasons for data aggregation were: 1) data augmentation (*i.e.* increase the number of available positive and negative regions for each TF); and 2) reduce potential biases from training on data from only one cell/tissue type (*i.e.* a model trained on A might not make useful predictions on B).

To explore the impact of data aggregation on transfer learning, we focused on GM12878 and K562 cells, which covered the largest number of TFs in the matrix. Using resolved regions in both cell types, we pre-trained a multi-task model for 50 TFs using matrix values from GM12878 cells (*i.e.* the model was not trained using K562 cell data). GM12878-specific resolved regions (*i.*e. regions resolved in GM12878 but not in K562 cells), and K562-specific resolved regions, were held-out for fine-tuning. Using the held-out data, we then trained single-task models, with and without transfer learning, for 35 of the 50 TFs used in the

pre-training step, as well as for an additional 76 TFs: 19 had resolved regions in both GM12878 and K562 cells (*i.e.* GM12878/K562 TFs); two in GM12878 but not in K562 cells (*i.e.* GM12878 TFs); and 55 in K562 but not in GM12878 cells (*i.e.* K562 TFs). Pre-training and fine-tuning on the same (*i.e.* GM12878) or on different cells (*i.e.* K562) did not impact transfer learning performance (average AUCPR = 0.835 vs. 0.814; Welch t-test, *p*-value = 0.202; **Fig. S6A**). However, models pre-trained and fine-tuned in the same cells benefited from transfer learning significantly more (average ΔAUCPR = 0.166 vs. 0.133; Welch t-test, *p*-value = 0.029; **Fig. S6B**). We attribute this to the poorer performance of single-task models trained from scratch on GM12878 cells compared to those trained on K562 cells for the 35 TFs from the multi-task model (average AUCPR = 0.656 vs. 0.713; Wilcoxon signed-rank test, *p*-value = 0.001; **Fig. S6D**). Taken together, transfer learning performance was stable across the two examined cell types.

We have included this control experiment in the revised version of the manuscript (see last paragraph of the results section “Transfer learning improves TF binding prediction”):

*Finally, since the TF binding matrix aggregates data across 52 cell and tissue types, we wondered if that would have an impact on transfer learning. We focused on GM12878 and K562 cells, which covered the largest number of TFs in the matrix.*

*Using resolved regions in both GM12878 and K562 cells, we pre-trained a*

*multi-model for 50 TFs common to both cell types, but only using matrix values from GM12878 cells (i.e. the multi-model was not trained using data from K562 cells).*

*GM12878-specific resolved regions (i.e. regions resolved in GM12878 but not in K562 cells), and K562-specific resolved regions, were held-out for fine-tuning. Using the held-out data, we then trained individual models, with and without transfer learning, for 35 of the 50 TFs used in the pre-training step (15 TFs were discarded because they had <250 resolved regions non-overlapping with multi-model regions left). We additionally trained individual models (with and without transfer learning) for 76 TFs with resolved regions in either GM12878 cells (2), K562 cells (55), or both (19).*

*Pre-training and fine-tuning on the same or on different cells did not impact transfer learning (Welch t-test, p-value = 0.202, NS;* ***Fig. S6A****), however, individual models pre-trained and fine-tuned in the same cells benefited from transfer learning significantly more (Welch t-test, p-value = 0.029;* ***Fig. S6B****), although differences were small (0.166 vs. 0.134). We attributed this to the poorer performance of individual models trained from scratch on GM12878 cells compared to those trained on K562 cells for the 35 multi-model TFs (average AUCPR = 0.656 vs. 0.713; Wilcoxon signed-rank test, p-value = 0.001;* ***Fig. S6D****). Taken together, these results rule out the introduction of a bias during the construction of the TF binding matrix due to aggregating the data in a cell and tissue type-agnostic manner.*

2. In many of the TFBS prediction models in the literature, size of the input regions are around 500bp to 1000bp. Did the authors try different sizes? If so, showing the performance of transfer learning regarding the input size would be informative. If not, elaborating on the selection of 200bp is needed in the manuscript.

The average size of the ENCODE DHS peak clusters from the UCSC Genome Browser [[4,5]](https://www.zotero.org/google-docs/?yxsd6u) was ~217 bp. We therefore chose to resize them to 200 bp. We have modified the methods section to clarify this point:

*Regions were resized, from an average length of ~217 bp, to 200 bp around the center of each DHS cluster using bedtools slop [55].*

As the reviewer points out, it has been reported in the literature that model performance increases with the length of the input sequence [[6,7]](https://www.zotero.org/google-docs/?O8F6nj), up to a maximum sequence length, after which the performance of the model plateaus or decays (in Leopard, such length is

~10,240 bp [[7]](https://www.zotero.org/google-docs/?hD7wcE)). Following the reviewer’s suggestion, we repeated the analysis from Fig. 8 (originally Fig. 7) using longer sequences, and confirmed the same trends: while the use of 500-bp and 1,000-bp long input sequences slightly improved model performance compared to using 200-bp long sequences, biologically-relevant transfer learning from TFs with the same binding mode as the target TF enhanced the results regardless of the length of the input sequence used.

Transfer learning performance using 200-bp long input sequences (Fig. 8):

Using 500-bp long input sequences (Fig. S8):

And using 1,000-bp long input sequences (Fig. S9):

The revised version of the manuscript has been modified to include the benefit of training on longer sequences:

*Similar results were obtained when the experiment was repeated, but using longer input sequences (****Figs. S8*** *and* ***S9****), as well as pre-training using five cofactors (****Fig. S10****).*

3. All individual models with transfer learning are actually trained further with a lower learning rate. For a fair comparison, the multi-task models also should be further trained with a lower learning rate. In addition, the single-task models without transfer learning should be first trained with a higher learning rate (as in multi-model) and then with a lower learning rate.

The transfer learning strategy used in this work (*i.e.* pre-training with a higher learning rate and fine-tuning with a smaller learning rate) is the current approach to transfer learning in the field of computer vision. The rationale is that features learned by the convolutional layers in the pre-training step could be “forgotten” if the fine-tuning step were performed with a high learning rate, rendering the transfer learning process futile.

We trained all models using the Adam optimizer [[8]](https://www.zotero.org/google-docs/?SZfhyk), which computes individual adaptive learning rates for different parameters of the neural network using first and second moments of the gradient. During the training process, the effective learning rate for each parameter varies dynamically between 0 and lambda. Hence, when training multi-task models it is not necessary to reduce the learning rate in later stages of the training process (*e.g.* using a learning rate scheduler), as this is automatically performed by the optimizer. Single-task models without transfer learning were trained with the same higher learning rate as the

multi-task model, and for the same reason, did not require reduction of the learning rate in later training stages.

4. The performance of the multi-task models are just shown in Figure 3-B. For the sake of fair and better comparison, the performance of the initial multi-task models can be added to the other figures too.

As noted by the reviewer, Fig. 3B is the only one wherein we show the performance of the multi-task model. This was possible because the 49 TFs shown in the figure had been used to train the multi-task model, and therefore, were part of the multi-task model output. This is not the case for other figures. For instance, Fig. 4 shows the transfer learning performance for HNF4A under different downsampling conditions. HNF4A was not amongst the 50 TFs used to train the multi-task model, and hence, the performance of the multi-task model cannot be shown. Related Figs. S2 to S5 show the same analysis for three TFs that had been used to train the multi-task model (*i.e.* JUND, MAX, and SP1), and SPI1, which was not amongst the 50 TFs used in the pre-training step (like HNF4A). Additional examples include Figs. 6 and 8 (and related Figs. S7 and S8-S10), wherein the target TF was only included in the pre-training step where indicated with the label “Target TF in multi-model”.

In general, multi-task models performed worse than single-task models (Fig. 3B; Table S2), particularly for underrepresented TFs. Given the focus of the manuscript on transfer learning, the performance of single-task models trained from scratch (*e.g.* Figs. 3 and 4), as well as that of transfer learning models from multi-task models pre-trained on random TFs (*e.g.* Figs. 6 and 8), provide a better baseline than the performance of multi-task models.

5. It is known that deep learning models need huge amounts of data to have decent learning. Similar to what the authors show, when there is less data, the performance of the models gets worse. In order to have a good negative control to measure baseline performance, the authors can use only PWMs to differentiate bound and unbound regions for individual TFs and calculate MCC. Particularly, a new PWM can be constructed ('trained') from the positive training data, while leaving out a subset of the positives. Using PWM-scanning with different thresholds on the left-out positives, versus negatives, the AUROC/AUPR can be calculated, or similarly to the deep learning models, the MCC can be compared (for example at the optimal threshold from the AUPR). This baseline is essential, to understand the performance (MCC) of both the multi-model and the fine-tuned models.

Following the reviewer’s suggestion, we applied STREME (version 5.3.0) [[9]](https://www.zotero.org/google-docs/?Ej9DXy) on the set of sequences used to train single-task models. We set the fraction of sequences held-out for *p*-value computation to 0 (option --hofract), the maximum PWM length to 21 bp (*i.e.* the length of CTCF profile as reported in JASPAR [[10]](https://www.zotero.org/google-docs/?HjtVbN), which was the longest in our dataset; option --maxw), and the number of output PWMs to 5 (option --nmotifs). PWM predictions were obtained using PWMScan (version 1.1.9) [[11]](https://www.zotero.org/google-docs/?6qHZE6).

We assessed two different scoring metrics on the set of 148 TFs from Fig. 3C: “best PWM score”, as suggested by the reviewer, and “sum occupancy score” (*i.e.* the sum of probabilities obtained from sliding the PWM along the forward and reverse complementary strands of a sequence [[12]](https://www.zotero.org/google-docs/?KZ6luY)). Independently of the scoring metric, the performance of the top *de novo* PWM (*i.e.* motif #1) was much better than the rest:

Moreover, even though the performance of both metrics were comparable, sum occupancy scores performed significantly better for the top *de novo* PWM:

|  | AUCPR |
| --- | --- |
| Best PWM score | 0.85 |
| Sum occupancy score | 0.86 |
| Wilcoxon signed-rank test | 4.0e-57 |

Taken together, subsequent analyses were based on the top *de novo* PWM and sum occupancy scores. Overall, performance of *de novo* PWMs (red) and transfer learning (orange) was comparable:

However, *de novo* PWMs performed better for TFs with small training datasets:
Performance of *de novo* PWMs was also better for TFs whose binding modes differed from those of the 50 TFs used to train the multi-task model:

The quality of results for *de novo* PWMs was somewhat expected, because the number of motif instances of the target TF was much higher in positive than negative sequences:

Hence, it should be relatively easy for *de novo* motif discovery tools to generate a PWM with which to discriminate positive from negative sequences. Moreover, as the reviewer noted, deep learning models require large datasets for successful training; it should be difficult for deep learning models to outperform *de novo* PWMs in this dataset unless they were provided with sufficiently large training datasets or initialized with adequate prior knowledge using transfer learning. We repeated the analysis for the 15 TFs from Figs. 8 and S10:

Fig. 8:

Fig. S10:

Biologically-relevant transfer learning performed comparably or better to *de novo* PWMs, except for zinc fingers KLF9 and ZNF740, and the basic helix-loop-helix factor SREBF2. As SREBF2 had only ~41.2 training and ~5.4 test sequences per data split, it should be regarded as an outlier.

The revised version of the manuscript describes the methods used to compute the baseline predictions from *de novo* PWMs for Figs. 3, 8, and S7-S10:

***De novo PWMs***

*For each TF and for each data split, we generated de novo PWMs by applying STREME (version 5.3.0) [62] on the train sequences. We set the fraction of sequences held-out for p-value computation to 0 (option --hofract), the maximum PWM length to 21 bp (i.e. the length of CTCF profile as reported in JASPAR, which was the longest in our dataset; option --maxw), and the number of output PWMs to 5 (option --nmotifs). We then computed the sum occupancy score (i.e. the sum of probabilities obtained from sliding the PWM along the forward and reverse complementary strands of a sequence [44]) of each test sequence using PWMScan (version 1.1.9) [63]. Performance was evaluated by means of AUCPR.*

It also reflects the performance of *de novo* PWMs in Fig. 3:

*By design of the TF binding matrix, the bound regions of each TF contained the motif of that TF, while the unbound regions could or could not contain the TF motif. This could make their classification too simple a task for the model. We therefore generated de novo PWMs for each TF to provide a more reliable baseline (****Methods****). Overall, transfer learning performance was comparable to that of de novo PWMs* (0.845 vs. 0.856; Wilcoxon signed-rank test, p-value = 0.667, NS)*, however, de novo PWMs outperformed transfer learning for TFs with small training datasets (****Fig. 3C****).*

And in Fig. 8:

*Finally, except for KLF9 and ZNF740, both zinc finger TFs, and SREBF2, which only had ~41.2 training and ~5.4 test sequences per data split (and hence should be considered an outlier), biologically-relevant transfer learning performed similar than or better to de novo PWMs (****Figs. 8*** *and* ***S8-S10****).*

6. The authors show that after the fine-tuning step, certain filters from the first convolutional layer represent binding motifs of the TF used for the fine-tuning. Can these filters be obtained by only using transfer learning? The authors can check the individual models without transfer learning whether the models can catch the binding sites of the related TF in their first convolutional layer.

Filters representing the motif of the target TF can be learned through transfer learning alone. In the results section “Interpretation of the transfer learning mechanism”, we demonstrated that an increased number of filters (six) became similar to the motifs of HNF4A with transfer learning compared to training from scratch (three; Table S4). Moreover, with transfer learning, the model learned the two distinct binding modes of HNF4A represented by the JASPAR profiles MA0114.4 and MA1494.1; when training from scratch, the model could only learn one of them (MA0114.4; Table S4).

Within the same results section, the JUND DeepLIFT/TF-MoDISco analysis covers the second point of the reviewer. The transfer learning model pre-trained on five cofactors of JUND yielded the canonical JASPAR profiles of CEBPB and SP1, both of which were amongst the cofactors used to train the multi-task model, as well as the motif of JUND (Fig. 9C). In contrast, for the transfer learning model pre-trained on five unrelated TFs,

TF-MoDISco could not identify the canonical motifs of JUND nor any of its cofactors (Table S4). Similarly, for the JUND model trained from scratch, none of the TF-MoDISco motifs corresponded to canonical motifs of JUND nor any of its cofactors (Table S4).

7. Measuring the performance of multi-task models can be elaborated, it is not clear from the text.

We apologize to the reviewer if the description of the methods in relation to measuring the performance of multi-task models was not sufficiently clear. We have clarified the description in the revised manuscript (see Methods section “Model performance”):

*To calculate the performance of a TF in the multi-model (****Fig. 3B****), we used the predictions on the test sequences by the output node in the multi-model corresponding to that TF.*

8. For the multi-task models, when the sample size of different classes are imbalanced, deep learning models tend to focus on the classes with the higher number of samples. This leads to poor performance on the classes with lower numbers of samples. In order to overcome this problem, using "class_weight" is suggested when training deep learning models. Did the authors try using the class_weight option when training the models?

We agree with the reviewer that class imbalance is likely responsible for the observed poor performance of underrepresented TFs in the multi-task model. As suggested by the reviewer, we tried using class weights, but the overall performance of the multi-task model worsened compared to training without class weights:

|  | With class weights | Without class weights |
| --- | --- | --- |
| True positives | 36068 | 19698 |
| False positives | 154391 | 6733 |
| True negatives | 408369 | 556027 |
| False negatives | 13272 | 29642 |
| AUCROC | 0.78 | 0.84 |
| AUCPR | 0.29 | 0.40 |
| Precision | 0.19 | 0.75 |
| Recall | 0.73 | 0.40 |
| Accuracy | 0.73 | 0.94 |

Multi-task model predictions using class weights:

Without using class weights:

Based on these results, we decided to not use class weights.

9. The authors mention that using DanQ architecture gives better performance, but throughout the whole paper another architecture is used. Can the authors elaborate on the selecting of the architecture used in this paper?

We apologize to the reviewer for the confusion. For a detailed explanation on our choice for model architecture, please refer to the following text included in the cover letter addressed to the editor:

*The manuscript focuses on the use of transfer learning for TF binding prediction, rather than performance relative to other methods. While performance is comparable*

*to current best practices in the field, the main impact of this report is to highlight the potential of transfer learning in the analysis of datasets of limited size. We build upon an existing model architecture that is well-established in the field. After demonstrating the benefits of transfer learning when pre-training with biologically-relevant TFs, we wondered if this observation was biased by our choice of model architecture. We therefore tested the system using the architecture of DanQ* [*[1]*](https://www.zotero.org/google-docs/?22Srqf)*.*

Accordingly, we have removed the sentence regarding the improvement in transfer learning performance when using the DanQ architecture:

*Consistent with previous studies [*[*24*](https://www.biorxiv.org/content/10.1101/2020.12.21.423873v1.full#ref-24)*,*[*46*](https://www.biorxiv.org/content/10.1101/2020.12.21.423873v1.full#ref-46)*], model performance was slightly better when using the DanQ architecture.*

10. Overall, if I understood correctly, when there is a TF with a similar binding mode to the final TF (or a cofactor of final TF) in the pre-trained multi-model, the transfer learning approach is more useful and increases the performance of the model significantly. This can be explained by the fact that convolutional filters have learned the binding motif of the final TF during the pre-training step. Indeed, the authors observe that freezing the convolution layers leads to decrease in the performance when applying transfer learning strategy. This in fact suggests that the most important part of the model, and of the

multi-to-finetuning strategy, are the convolutional layers. These results suggest that inserting PWMs to the convolutional layers prior to training the 1-TF model may have an huge impact on the performance of the model and may even outperform the incremental effect of transfer learning. Therefore, we suggest testing a 1 TF model using Jaspar PWMs as convolutional filters. If such a strategy works as efficiently, then can we conclude that the major contribution of the multi-model step (and the transfer learning), is to set the priors correctly? This seems to be a critical thing to assess, given that this aspect forms the core of the paper/novelty.

We agree with the reviewer that convolutional layers play an important role, if not the most important, in the fine-tuning step. We trained single-task models wherein we initialized the first layer convolutional filters with a combination of non-redundant JASPAR profiles [[10]](https://www.zotero.org/google-docs/?tG9G6v) and *de novo* PWMs. We converted PWMs to filter weights as described in the DanQ manuscript [[1]](https://www.zotero.org/google-docs/?uobkeQ): PWMs were resized to 19 bp and we subtracted 0.25 from the probability of each nucleotide at each position. We focused the analysis on the 10 TFs from Fig. 8 (originally Fig. 7) with an intermediate (1,000-10,000) and small (<1,000) number of bound regions: NR2C2 and VDR (nuclear receptors); ATF7 and NFE2L1 (basic leucine zippers); MNT and SREBF2 (basic helix-loop-helix factors); ETV4 and ERG (tryptophan cluster factors); and KLF9 and ZNF740 (zinc fingers). Having >10,000 bound regions, HNF4A, JUND, MAX, SPI1, and SP1 were considered too easy to train and predict and were therefore excluded from the analysis. As reported in the DanQ manuscript, initialization with JASPAR profiles and *de novo* PWMs (yellow) resulted in better performance compared to training from scratch (red). However, the performance was inferior to that of biologically-relevant transfer learning (blue).

To further confirm the central role of convolutional layers in the transfer learning process, instead of transferring the convolutional and fully-connected layers (except the output layer), we either transferred the weights of the first (light blue) or all convolutional layers (green); *i.e.* we did not transfer the weights of the fully-connected layers. Doing so only resulted in slightly worse performance compared to the original transfer learning strategy, particularly in the second case where we transferred all the convolutional layers.

We have included this experiment in the revised version of the manuscript (see the last paragraph of the results section “Interpretation of the transfer learning mechanism”):

*Finally, to further confirm the central role of convolutional layers, particularly filters, in the transfer learning process, we focused on the 10 TFs from* ***Fig. 8*** *with an intermediate (1,000-10,000) and small (<1,000) number of bound regions. We trained individual models with transfer learning wherein we transferred the weights of either the first or all three convolutional layers (i.e. we did not transfer the weights from the fully-connected layers). Doing so only slightly worsened performance compared to the original transfer learning strategy, particularly when we transferred all three convolutional layers (****Fig. S11****). We also explored the importance of convolutional filters by initializing individual models with a combination of non-redundant profiles from the JASPAR database and de novo PWMs (****Methods****). As previously reported [46], filter initialization with JASPAR profiles, and, in our case, de novo PWMs, improved model performance compared to training from scratch, however, performance was inferior to that of biologically-relevant transfer learning (****Fig. S11****).*

**Reviewer #2**: The authors investigate the problem of learning TF binding predictions from Chip-seq data for many different TFs using Deep neural networks with transfer learning. This paper is very timely as the use of transfer learning is becoming very prominent and the observations are of interest. The paper is well written and the experiments are sound. The authors cite a preprint (ref 24, which is got published in Nature Machine Intelligence early 2021), which has similairity in that a similar transfer learning strategy is used. However, I personally find that there is not a lot of overlap between the two papers, and that the manuscript here is much more interesting for biologists and computational biologists. In

particular the analysis concerning the similarity of binding sites and protein domains, is something which likely extrapolates to related problems, such as the prediction of RNA-binding proteins.

We thank the reviewer for the comments and for drawing our attention to the recent publication of AgentBind in Nature Machine Intelligence.

1. The authors propose a transfer learning approach to predict TFBS from DNA sequence. Their design is very similar to the newly published work AgentBind, "Zheng et.al., nature machine intelligence, 2021", which they also mention in their paper by stating that they corroborate the findings of Zheng and colleagues. I would find it helpful if the authors would state differences to their study and the other work more clearly (number of TFs, analysis goals etc.).

We agree with the reviewer that it would be helpful to specify the similarities and differences between the two works. Briefly, AgentBind aims to identify distinctive contextual sequence characteristics between bound and unbound instances of TF motifs. The authors focus the analysis on 38 TFs of the GM12878 cell line by first pre-training a multi-task model on the DeepSEA dataset [5] (*i.e.* >4M 1-kb long sequences annotated with a total of 919 ChIP-seq and DNase-seq profiles from ENCODE [[4]](https://www.zotero.org/google-docs/?F6Kpbf) and the Epigenomics Roadmap Project [[13]](https://www.zotero.org/google-docs/?kNYH5z) across multiple cell lines), followed by fine-tuning of 38 single-task models (one for each TF), previously initialized with the pre-trained model, using ChIP-seq data from GM12878 cells.

While AgentBind is an important contribution to the field, we agree with the reviewer that its publication does not detract from the potential interest to the reader in the work described in this manuscript.

There are key differences between the dataset construction, design, and goals between the two works:

- In AgentBind, both the positive and negative training sequences contain the motif of the target TF. This is different from our approach, wherein the positive sequences contain the motif (*i.e.* to focus on direct binding events), whereas the negative sequences may or may not contain the motif of the target TF.
- Moreover, in AgentBind, the authors focus the analysis on 38 TFs in a fixed cell line (*i.e.* GM12878), whereas our analysis covers 148 TFs (15 TFs were discarded because they did not have enough regions with which to train) and combines data from 52 cell and tissue types in a cell and tissue type-agnostic manner.
- In AgentBind, only the weights from the convolutional layers are transferred, while we transfer the weights from both the convolutional and fully-connected layers (except the output layer).
- Finally, we assessed multiple pre-training strategies (*e.g.* pre-training on 50 TFs, on five cofactors, etc.), whereas AgentBind uses a fixed pre-trained model for all analyses.

We have modified the manuscript to highlight these similarities/differences. In the results section “Transfer learning improves TF binding prediction”:

*Zheng and colleagues recently applied transfer learning to identify distinctive contextual sequence characteristics of bound and unbound instances of TF motifs* [*[24]*](https://www.zotero.org/google-docs/?GvmG73)*. Their transfer learning framework, named AgentBind, included a pre-training step, wherein a multi-task model (hereafter referred to as multi-models) was trained on the DeepSEA dataset* [*[43]*](https://www.zotero.org/google-docs/?HKa2U4)*, and a fine-tuning step, wherein single-task models (hereafter referred to as individual models) for 38 individual TFs of the GM12878 cell line were trained after previous initialization of the convolutional layers with weights from the multi-model. AgentBind outperformed models trained from scratch (i.e. without transfer learning), particularly for TFs with little ChIP-seq data. To corroborate these results, we implemented a similar two-step transfer learning strategy for TF binding prediction (****Methods****;* ***Fig. 3A****), however, in the fine-tuning step, both the convolutional and fully-connected layers (except the output layer) of individual models were initialized with the multi-model weights, after which the training continued at a lower learning rate.*

2. Regarding creating the data, they define three different states based on the binding status of TFs in DNase I hypersensitive sites (DHS). They define state "null" when the binding of the TF to the DHS could not be resolved, which is different than state "0" where DHS was accessible but not bound by the TF. The number of null cases they identified in their data is more than 80%. Since this number was really high, they decided to use a cosine-base similarity metric combined with MCC (Mathew's correlation coefficient) for their assessment of transfer learning. They show the AUROC and AUCPR on the MCC values. But, I'm not sure why they didn't show the AUROC and AUCPR results directly on the output of their model. Was it because they formulated their problem into three states, instead of two(bound and unbound), or they wanted to avoid showing these metric due to the imbalance nature of the data? Perhaps they could elaborate on these points and otherwise show also AUCPR to show that their general conclusions hold as well using this established metric.

We apologize to the reviewer for the lack of clarity in our approach, which could have led to their confusion. The reviewer is correct that we defined three different TF binding states (open/bound = 1; open/unbound = 0; unresolved = null). However, we did not use a combination of cosine similarity and MCC for the assessment of transfer learning.

Regarding the use of the cosine similarity metric: As indicated by the reviewer the TF binding matrix contained >80% of unresolved regions. This observation led to concerns about the sparsity of the matrix and whether it had captured known TF-TF functional associations (*e.g.* dimeric complexes or TF cooperativity). We therefore computed pairwise cosine similarities between the binding vectors of all TFs in the matrix as a measure of correlation (Fig. 2; see third paragraph of the results section “A sparse matrix of TF binding events across accessible genomic regions”). Using these cosine similarities, we then define sets of cofactors with which to train multi-task models (see second paragraph of the results section “Biologically-relevant prior knowledge improves transfer learning”).

Regarding the use of the MCC metric: For evaluating binary classification tasks, MCC is a more reliable metric than AUCROC or AUCPR. It returns a high score only if the classifier achieves good results in all four categories of the confusion matrix (true positives, false negatives, true negatives, and false positives), proportionally to both the number of positives and negatives [[14]](https://www.zotero.org/google-docs/?0GcHle). However, unlike AUCROC or AUCPR, it has the disadvantage of requiring an arbitrary threshold with which to define positive and negative predictions.

In response to the reviewer’s suggestion, and to avoid any confusion to the reader, we have replaced the MCC with the AUCPR metric. Noteworthy, this change does not affect the interpretation of the results as both metrics were highly correlated.

3. It is not clear for the different experiments they did, what the exact input TFs were. For example. In page 6, lines 18-21, where do they get the additional 99 TFs from? How come they were not used in training the multi-model? It would be helpful if the authors define different input sets that are used for the experiments and then refer to them in a systematic way. in particular it needs to be made very clear, if the same TF was used in the multi-task step or not.

Following the reviewer's suggestion, we have highlighted in the manuscript the list of TFs that were used for each experiment. To train the initial multi-task model (see the first paragraph of the results section “Transfer learning improves TF binding prediction”), we used 50 TFs with the highest number of resolved regions in the TF binding matrix. This model was used to initialize single-task models for 49 of those TFs (Fig. 3B). ARNT was excluded from the analysis because it did not have enough regions left for training that did not overlap with the pre-training step. Next, we used the pre-trained multi-task model to initialize single-task models for 99 additional TFs that were not amongst the 50 TFs used in the pre-training step and had a sufficiently large number of regions non-overlapping with the pre-training step.

For Fig. 4, we modified the text to indicate more clearly which TFs were used in the pre-training step:

*We focused on five TFs from five different families: HNF4A (a nuclear receptor), JUND (a basic leucine zipper), MAX (a basic helix-loop-helix factor), SPI1 (a tryptophan cluster factor), and SP1 (a C2H2 zinc finger). Of these, JUND, MAX, and SP1 were amongst the 50 TFs used to train the multi-model.*

For Figs. 6 and 8 (originally Fig. 7), we indicate whether the target TF was used in the

pre-training step, and report results for both cases. Finally, for Fig. 7 (originally Fig. 8), the target TF was not used in the pre-training step, as noted in the text:

*For this set of experiments, the target TF was not included in the pre-training step, and we fixed the training datasets sizes of multi-models and individual models to*

∼*40,000 and* ∼*2,000 regions, respectively.*

4. The usage of the term "transfer learning" is sometimes a bit unclear in the context. In page 8, line 60 they state "... inadequate set of weights with which to initialize the individual model...". Is it just initializing or really transfer and freezing the weights? In other words, to this point it's not clear if they modify the weights after initializing using the

multi-model step. But, when reading the "transfer learning" section in "Methods", they state that in the fine-tuning step they either train the entire model or just the fully connected layers. Which again it is not clear which case they use. It needs to be made very clear what is freezed when using transfer learning and what not. It should be straightforward to introduce a different learning setups to differentiate between those modes and then properly refer to them in the text.

We apologize to the reviewer for the confusion. Single-task models were initialized from a pre-trained model, after which all layers were fine-tuned using a lower learning rate. The

fine-tuning step applied to both convolutional and fully-connected layers. However, there was one experiment wherein to confirm if the refinement of convolutional filters in the fine-tuning step was responsible for the improvement in transfer learning performance, we applied an alternative fine-tuning strategy. Specifically, we froze the convolutional layers of the

pre-trained multi-task model and fine-tuned only the fully-connected layers (*i.e.* no fine-tuning of the convolutional layers was allowed):

*To confirm that the refinement of convolutional filters in the fine-tuning step was responsible for the improvement in model performance by transfer learning, we applied an alternative fine-tuning strategy: we froze the convolutional layers of the pre-trained multi-model and trained only on the fully-connected layers (i.e. no refinement of convolutional layer filters was allowed).*

We have modified the methods section to clarify the transfer learning strategy:

*[...] Unless otherwise specified, in the fine-tuning step, we trained the entire model including the convolutional and fully-connected layers.*

1. [Quang D, Xie X. DanQ: a hybrid convolutional and recurrent deep neural network for](https://www.zotero.org/google-docs/?1CbOQR) [quantifying the function of DNA sequences. Nucleic Acids Res. Oxford Academic;](https://www.zotero.org/google-docs/?1CbOQR) [2016;44:e107–e107.](https://www.zotero.org/google-docs/?1CbOQR)
2. [Zheng A, Lamkin M, Zhao H, Wu C, Su H, Gymrek M. Deep neural networks identify](https://www.zotero.org/google-docs/?1CbOQR) [sequence context features predictive of transcription factor binding. Nat Mach Intell. Nature](https://www.zotero.org/google-docs/?1CbOQR) [Publishing Group; 2021;3:172–80.](https://www.zotero.org/google-docs/?1CbOQR)
3. [Lan G, Zhou J, Xu R, Lu Q, Wang H. Cross-Cell-Type Prediction of TF-Binding Site by](https://www.zotero.org/google-docs/?1CbOQR) [Integrating Convolutional Neural Network and Adversarial Network. Int J Mol Sci.](https://www.zotero.org/google-docs/?1CbOQR) [Multidisciplinary Digital Publishing Institute; 2019;20:3425.](https://www.zotero.org/google-docs/?1CbOQR)
4. [Dunham I, Kundaje A, Aldred SF, Collins PJ, Davis CA, Doyle F, et al. An integrated](https://www.zotero.org/google-docs/?1CbOQR) [encyclopedia of DNA elements in the human genome. Nature. Nature Publishing Group;](https://www.zotero.org/google-docs/?1CbOQR) [2012;489:57–74.](https://www.zotero.org/google-docs/?1CbOQR)
5. [Lee CM, Barber GP, Casper J, Clawson H, Diekhans M, Gonzalez JN, et al. UCSC](https://www.zotero.org/google-docs/?1CbOQR) [Genome Browser enters 20th year. Nucleic Acids Res. Oxford Academic; 2020;48:D756–61.](https://www.zotero.org/google-docs/?1CbOQR)
6. [Zhou J, Troyanskaya OG. Predicting effects of noncoding variants with deep](https://www.zotero.org/google-docs/?1CbOQR) [learning–based sequence model. Nat Methods. Nature Publishing Group; 2015;12:931–4.](https://www.zotero.org/google-docs/?1CbOQR)
7. [Li H, Guan Y. Fast decoding cell type–specific transcription factor binding landscape at](https://www.zotero.org/google-docs/?1CbOQR) [single-nucleotide resolution. Genome Res. 2021;31:721–31.](https://www.zotero.org/google-docs/?1CbOQR)
8. [Kingma DP, Ba J. Adam: A Method for Stochastic Optimization. ArXiv14126980 Cs](https://www.zotero.org/google-docs/?1CbOQR) [[Internet]. 2017 [cited 2020 Jul 10]; Available from: http://arxiv.org/abs/1412.6980](https://www.zotero.org/google-docs/?1CbOQR)
9. [Bailey TL. STREME: accurate and versatile sequence motif discovery. Bioinformatics](https://www.zotero.org/google-docs/?1CbOQR) [[Internet]. 2021 [cited 2021 May 12]; Available from:](https://www.zotero.org/google-docs/?1CbOQR) [https://doi.org/10.1093/bioinformatics/btab203](https://www.zotero.org/google-docs/?1CbOQR)
10. [Fornes O, Castro-Mondragon JA, Khan A, van der Lee R, Zhang X, Richmond PA, et al.](https://www.zotero.org/google-docs/?1CbOQR)

[JASPAR 2020: update of the open-access database of transcription factor binding profiles.](https://www.zotero.org/google-docs/?1CbOQR) [Nucleic Acids Res. Oxford Academic; 2020;48:D87–92.](https://www.zotero.org/google-docs/?1CbOQR)

11. [Ambrosini G, Groux R, Bucher P. PWMScan: a fast tool for scanning entire genomes with](https://www.zotero.org/google-docs/?1CbOQR) [a position-specific weight matrix. Bioinformatics. 2018;34:2483–4.](https://www.zotero.org/google-docs/?1CbOQR)
12. [Ambrosini G, Vorontsov I, Penzar D, Groux R, Fornes O, Nikolaeva DD, et al. Insights](https://www.zotero.org/google-docs/?1CbOQR) [gained from a comprehensive all-against-all transcription factor binding motif benchmarking](https://www.zotero.org/google-docs/?1CbOQR) [study. Genome Biol. 2020;21:114.](https://www.zotero.org/google-docs/?1CbOQR)
13. [Kundaje A, Meuleman W, Ernst J, Bilenky M, Yen A, Heravi-Moussavi A, et al. Integrative](https://www.zotero.org/google-docs/?1CbOQR) [analysis of 111 reference human epigenomes. Nature. Nature Publishing Group;](https://www.zotero.org/google-docs/?1CbOQR) [2015;518:317–30.](https://www.zotero.org/google-docs/?1CbOQR)
14. [Chicco D, Jurman G. The advantages of the Matthews correlation coefficient (MCC) over](https://www.zotero.org/google-docs/?1CbOQR) [F1 score and accuracy in binary classification evaluation. BMC Genomics. 2020;21:6.](https://www.zotero.org/google-docs/?1CbOQR)

**Second round of review**

**Reviewer 1**

The authors have adequately addressed all our comments, congratulations with the revised manuscript.

**Reviewer 2**

The authors have sufficiently addressed my concerns and improved the description of the different setups. I think that the revised manuscript is much better readable and presents the interesting results well.
